# STARD10 promotes progression of HER2+ breast cancer and intracellular lipid metabolism via the cAMP/PKA/CREB1 signaling axis

**DOI:** 10.1080/15384047.2026.2688544

**Published:** 2026-06-15

**Authors:** Siyao Liu, Jing Gao, Yichen Niu, Xinnan Dong, Zhiming Zhang, Xiao Teng, Zhaorong Li, Siyan Zhang, Yong Meng, Ming-Qing Gao

**Affiliations:** a Northwest University First Hospital, Xi’an, Shaanxi, People's Republic of China; b College of Life Sciences, Northwest University, Xi’an, Shaanxi, People's Republic of China; c School of Medicine, Northwest University, Xi’an, Shaanxi, People's Republic of China

**Keywords:** STARD10, HER2+ breast cancer, cAMP/PKA/CREB1, tumor progression, lipid droplets

## Abstract

**Background:**

Although targeted therapies have improved clinical outcomes, HER2+ breast cancer remains a significant clinical challenge due to its aggressive behavior and unfavorable prognosis. Emerging evidence indicates that dysregulated lipid metabolism plays a critical role in tumorigenesis and metastasis, suggesting that targeting lipid metabolism may represent a promising therapeutic strategy. STARD10, a lipid transport protein, plays a pivotal role in regulating lipid metabolism. However, its function in mediating lipid metabolism and tumor progression in HER2+ breast cancer remains unclear.

**Methods:**

The expression level and prognostic relevance of STARD10 in HER2+ breast cancer were analyzed using public databases and clinical cohorts. CCK-8, EdU, colony formation, transwell, LD540, and Nile Red staining assays were performed in SKBR3 and HCC1954 cells. Subcutaneous implantation and tail vein injection were performed to evaluate the effects of STARD10 overexpression on tumor growth and lung metastasis in vivo. The mechanism was validated by RNA-seq and Western blotting.

**Results:**

STARD10 expression was upregulated in HER2+ breast cancer tissues and was significantly correlated with poor prognosis. Functionally, STARD10 overexpression enhanced HER2+ breast cancer cell proliferation, migration, invasion, and lipid droplets accumulation. Moreover, STARD10 overexpression markedly accelerated tumor growth and lung metastasis in vivo. Mechanistically, STARD10 was found to drive malignant phenotypes via activation of the cAMP/PKA/CREB1 signaling axis.

**Conclusion:**

STARD10 promotes malignant progression of HER2+ breast cancer and lipid droplets accumulation by activating the cAMP/PKA/CREB1 pathway. These findings suggest that STARD10 and the cAMP/PKA/CREB1 signaling axis as potential therapeutic targets for the treatment and prevention of HER2+ breast cancer.

## Graphical abstract

**Figure:**
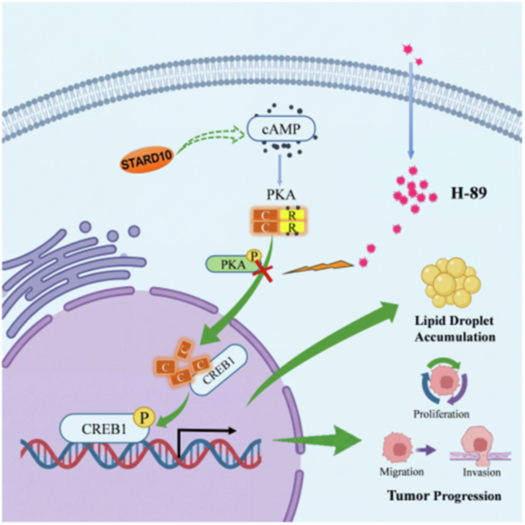

Schematic illustration of the STARD10-cAMP/PKA/CREB1 axis in promoting HER2+ breast cancer progression and intracellular lipid metabolism.

## 1. Introduction

Breast cancer remains a major global health problem, affecting women of all ages worldwide.^1^ The female mortality rate of breast cancer is 15.4%, representing the leading cause of cancer-related death among women.^2^ ^,^ ^3^ The development of breast cancer is driven by a complex interplay of genetic, hormonal, environmental, and lifestyle factors, yet its underlying pathogenesis remains incompletely understood.^4^ ^,^ ^5^ Based on the expression of the estrogen receptor, the progesterone receptor, and human epidermal growth factor receptor 2 (HER2), breast cancer is broadly classified into four molecular subtypes: Luminal, HER2+ , Basal-like, and TNBC.^6^ ^,^ ^7^

Human epidermal growth factor receptor 2-positive (HER2+) breast cancer, which accounts for 15%-20% of all breast cancer cases, is characterized as an aggressive subtype driven by overexpression of the HER2 receptor tyrosine kinase.^8^ ^,^ ^9^ HER2 (ERBB2) overexpression drives aberrant tumor cell proliferation and malignant progression, leading to faster disease progression and poorer prognosis in patients with HER2+ breast cancer than in those with HER2- breast cancer.^9-11^ Although targeted therapies such as trastuzumab have significantly improved clinical outcomes, HER2+ breast cancer continues to be associated with unfavorable prognosis in a subset of patients.^12^ ^,^ ^13^ Recent studies suggest that metabolic adaptation contributes significantly to cancer progression, particularly lipid metabolic reprogramming.^14^ Therefore, identifying key regulators of lipid metabolism may offer novel therapeutic opportunities for this aggressive breast cancer subtype.

STARD10, a member of the steroidogenic acute regulatory protein family, possesses a steroidogenic acute regulatory protein-related lipid transfer domain that facilitates the transport of phosphatidylcholine and phosphatidylethanolamine between intracellular membranes.^15^ ^,^ ^16^ Clinical data reveal that STARD10 expression is elevated in 35% of primary breast carcinomas, 64% of human breast cancer cell lines, and murine mammary tumors.^17^ ^,^ ^18^ The activity of STARD10 is negatively regulated by casein kinase II-mediated phosphorylation at serine 284.^19^ ^,^ ^20^ Aberrant expression of this gene may induce cellular transformation and tumorigenesis via dysregulation of lipid-related pathways.^21^ Importantly, STARD10 is highly expressed in HER2+ breast cancer and is co-expressed with ERBB2 to synergistically promote tumorigenesis.^19^ Despite these findings, the exact molecular mechanisms by which STARD10 underlies disease progression and lipid metabolic reprogramming in HER2+ breast cancer remain elusive.

Our study aimed to investigate the biological functions of STARD10 in HER2+ breast cancer and to elucidate the potential role of the cAMP/PKA/CREB1 signaling pathway in mediating STARD10-driven tumor progression and lipid metabolism. Understanding this mechanism may provide novel therapeutic strategies for HER2+ breast cancer treatment.

## 2. Results

### 2.1. Analysis of the clinical relevance of STARD10 expression with breast cancer

**Figure 1.**
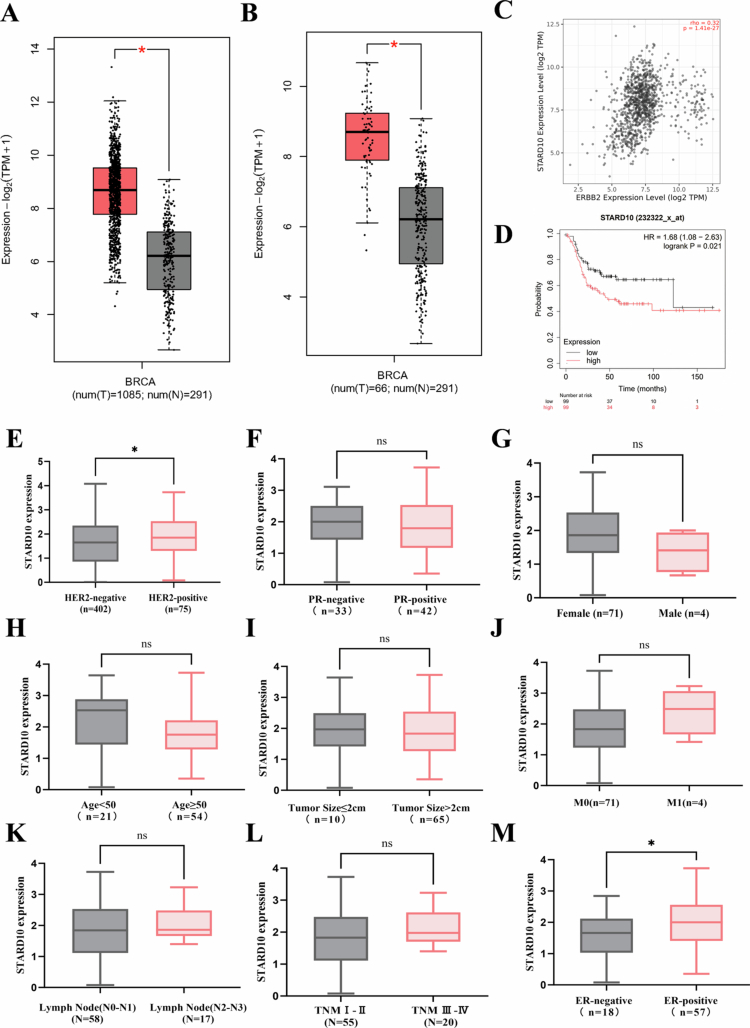
Analysis of the Clinical Relevance of STARD10 Expression with Breast Cancer. **(A, B)** STARD10 expression levels in breast cancer, HER2+ , and normal breast tissues from in TCGA database. **(C)** Correlation analysis between STARD10 expression and ERBB2 from TCGA database. **(D)** Overall survival (OS) curve of HER2+ breast cancer patients with low and high STARD10 expression from TCGA cohort. **(E)** STARD10 expression levels in HER2- breast cancer and HER2+ breast cancer tissues analyzed from GEO database. **(F-M)** Correlation analysis between STARD10 and progesterone receptor status, gender, age, maximum tumor diameter, distant metastasis, lymph node metastasis, tumor-node-metastasis stage, and estrogen receptor in HER2+ breast cancer samples from the GEO database. *P < 0.05; ns, not significant.

Based on database analysis, we found that STARD10 expression was significantly higher in breast tumor tissues (*n* = 1085) than in normal breast tissues (*n* = 291) (Figure 1A and *Supplementary Fig. S1*), and this elevated expression was particularly pronounced in the HER2+ breast cancer subtype (*n* = 66) (Figure 1B). Validation at the protein level in vitro similarly demonstrated that STARD10 expression was significantly upregulated in HER2+ breast cancer cells compared with normal mammary epithelial cells (*Supplementary Fig. S2*). To further investigate the potential association between STARD10 and HER2, we analyzed the expression profile of STARD10 in SKBR3, a representative HER2+ breast cancer cell line, using the Human Protein Atlas database. The results confirmed high STARD10 expression in the SKBR3 cell line (*Supplementary Fig. S3*). Furthermore, correlation analysis based on the TIMER2.0 database revealed a significant positive correlation between STARD10 and ERBB2 expression (Figure 1C). These findings are highly consistent with a previous study, which reported that STARD10 is highly expressed in HER2+ breast cancer and acts synergistically with the HER2 receptor.^17^

Analysis of the TCGA cohorts revealed that STARD10 expression was significantly higher in HER2+ patients (*n* = 75) than in HER2- patients (*n* = 402) (Figure 1E). Guided by this finding, we conducted a clinicopathological analysis of a retrospective cohort comprising 75 cases of HER2+ breast cancer from the GEO database. The results showed that STARD10 expression was not significantly correlated with progesterone receptor status, gender, age, maximum tumor diameter, distant metastasis, lymph node metastasis, or tumor-node-metastasis stage (Figures 1F−L). Notably, a significant positive correlation was observed between STARD10 expression and estrogen receptor status (Figure 1M), which may be attributed to the crosstalk between the HER2 and estrogen receptor signaling pathways.^22^ Furthermore, Kaplan-Meier analysis was performed to evaluate the association between STARD10 expression and HER2+ breast cancer prognosis. The results indicated that patients with high STARD10 expression exhibited shorter overall survival (Figure 1D), suggesting that STARD10 may serve as a biomarker for poor prognosis in HER2+ breast cancer.

### 2.2. STARD10 overexpression promotes progression of HER2+ breast cancer and intracellular lipid droplets synthesis

**Figure 2.**
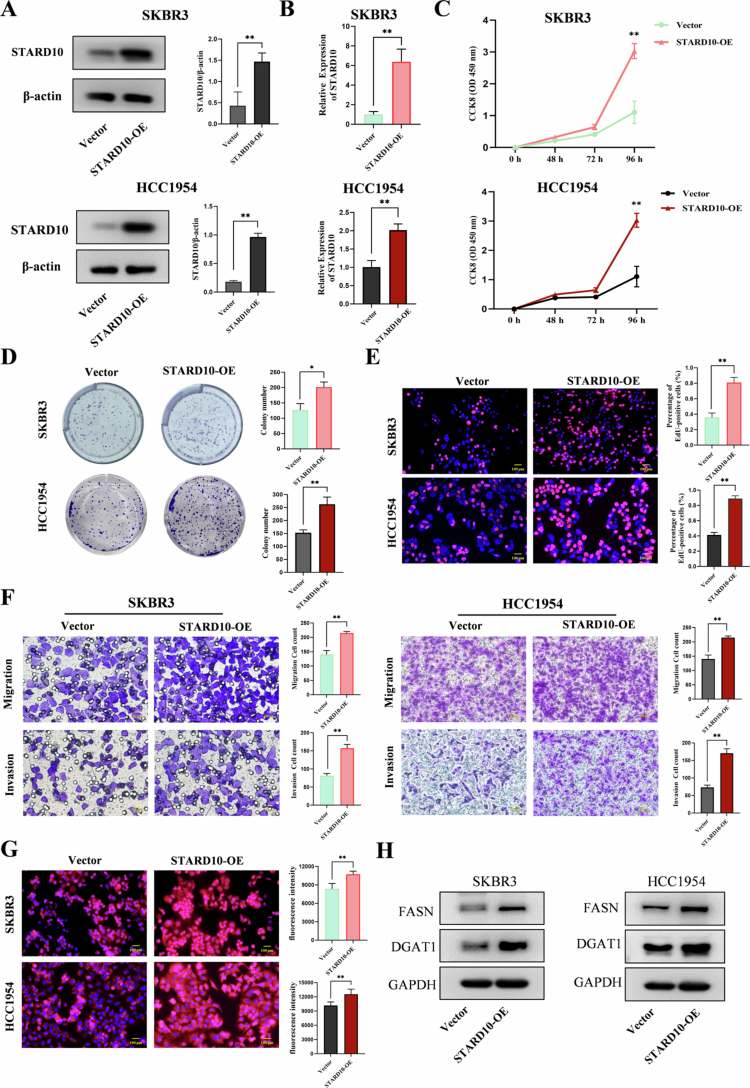
STARD10 overexpression promotes progression of HER2+ Breast Cancer and intracellular lipid droplets synthesis. **(A, B)** Western blot and q-PCR analysis for STARD10 expression in SKBR3 and HCC1954 cell lines transduced with lentiviral vectors. (**C-E)** CCK-8, colony formation and EdU assays were performed to evaluate the proliferation capacity of STARD10-overexpressing SKBR3 and HCC1954 cells. Scale bar = 100 μm. **(F)** Transwell assays were performed to evaluate cell migration and invasion capabilities of STARD10-overexpressing SKBR3 and HCC1954 cells. Scale bar = 50 μm. **(G**) Representative images of intracellular lipid droplets in STARD10-overexpressing SKBR3 and HCC1954 cells stained with LD540. The corresponding quantification shows the fluorescence intensity of lipid droplets. Scale bar = 100 μm. **(H**) Western blot analysis of FASN and DGAT1 expression levels in STARD10-overexpressing SKBR3 and HCC1954 cells. *n* = 3. *P < 0.05, **P < 0.01.

To elucidate the biological function of STARD10 in HER2+ breast cancer, we successfully established stable STARD10-overexpressing SKBR3 and HCC1954 cell lines by lentiviral transduction (*Supplementary Fig. S4*), and the overexpression was verified at both protein and mRNA levels (Figure 2A and B). Functionally, STARD10 overexpression significantly enhanced the proliferation of both SKBR3 and HCC1954 cells (Figure 2C−E). Transwell assays further demonstrated that STARD10 overexpression promoted the migratory and invasive capabilities of SKBR3 and HCC1954 cells (Figure 2F). Furthermore, STARD10 overexpression enhanced the formation of 3D spheroids (*Supplementary Fig. S5*). Given that lipid droplets represent a major quantitative component of cellular lipids,^23^ our results showed that STARD10 overexpression significantly increased lipid droplets content in SKBR3 and HCC1954 cells (Figure 2G and *Supplementary Fig. S6*). Meanwhile, Western blot analysis revealed that STARD10 overexpression markedly enhanced the protein expression of FASN and DGAT1 (Figure 2H and *Supplementary Fig. S7*). Collectively, these results demonstrate that STARD10 serves as a key regulator of lipid droplets formation in HER2+ breast cancer.

### 2.3. STARD10 knockout inhibits the progression of HER2+ breast cancer and intracellular lipid droplets synthesis

**Figure 3.**
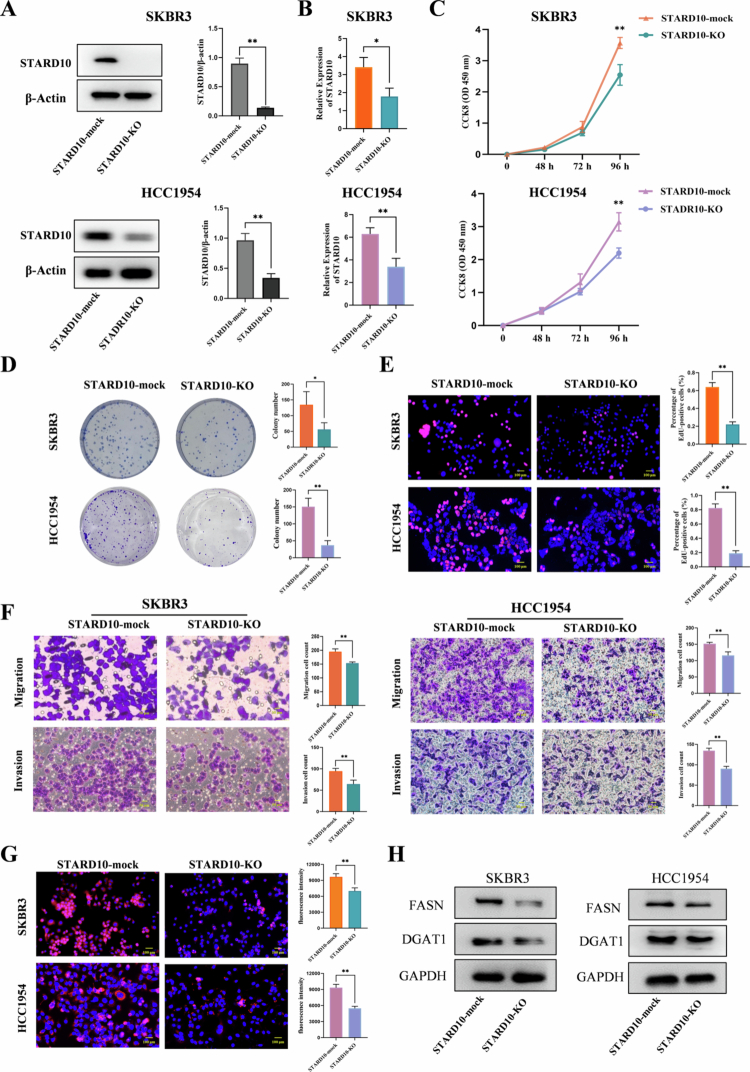
STARD10 knockout inhibits the progression of HER2+ Breast Cancer and intracellular lipid droplets synthesis. **(A, B)** Western blot and q-PCR analysis for STARD10 expression in SKBR3 and HCC1954 cell lines edited using CRISPR/Cas9. (**C-E)** CCK-8, colony formation and EdU assays were performed to evaluate the proliferation capacity of stable STARD10-knockout SKBR3 and HCC1954 cells. Scale bar = 100 μm. **(F)** Transwell assays were performed to evaluate cell migration and invasion capabilities of stable STARD10-knockout SKBR3 and HCC1954 cells. Scale bar = 50 μm. **(G**) Representative images of intracellular lipid droplets in stable STARD10-knockout SKBR3 and HCC1954 cells stained with LD540. The corresponding quantification shows the fluorescence intensity of lipid droplets. Scale bar = 100 μm. **(H**) Western blot analysis of FASN and DGAT1 expression in STARD10-knockout SKBR3 and HCC1954 cells. *n* = 3. *P < 0.05, **P < 0.01.

Complementary loss-of-function studies were conducted to further investigate the biological significance of STARD10 in HER2+ breast cancer cells. Using CRISPR/Cas9 gene editing technology, we established stable STARD10-knockout SKBR3 and HCC1954 cell lines, in which the protein and mRNA expression levels of STARD10 were significantly decreased (Figure 3A and B). The CCK-8, colony formation and EdU assays demonstrated that STARD10 knockout significantly inhibited the proliferation of SKBR3 and HCC1954 cells (Figure 3C−E). Additionally, STARD10 knockout suppressed the migration and invasion of SKBR3 and HCC1954 cells (Figure 3F) and significantly reduced lipid droplets content (Figure 3G and H; *Supplementary Figs. S8 and S9*). Notably, no statistically significant difference was observed in 3D spheroid formation between groups (*Supplementary Fig. S10*), which may be attributed to the relatively low endogenous STARD10 expression level in these cell lines.

### 2.4. STARD10 promotes the growth and metastasis of HER2+ breast cancer cells *in vivo*

**Figure 4.**
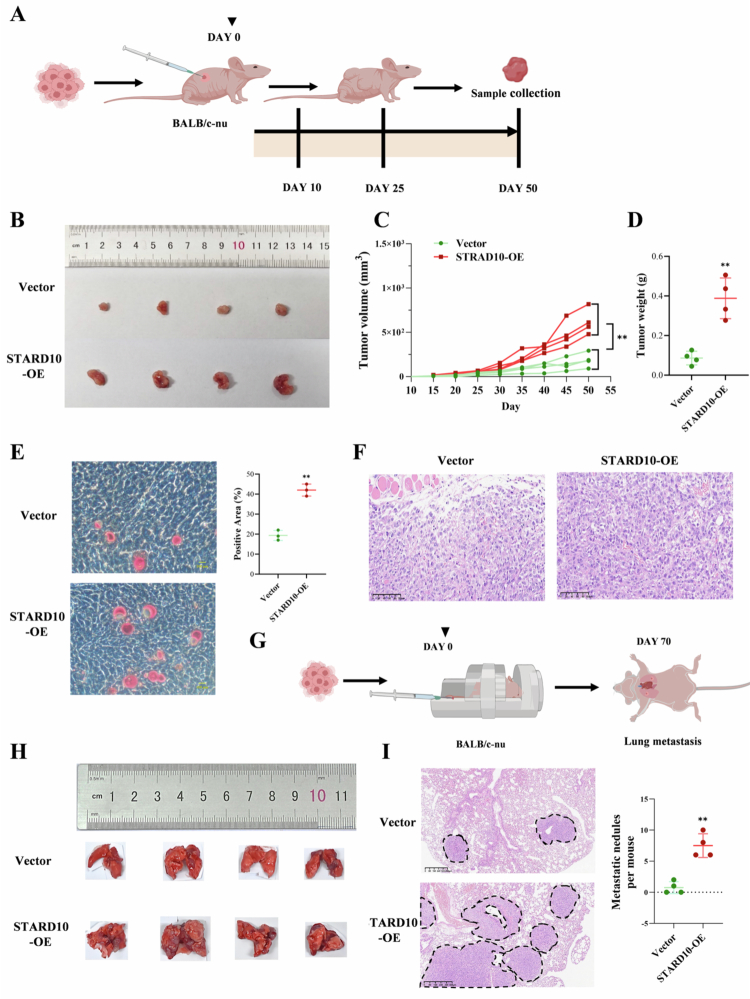
STARD10 promotes the growth and metastasis of HER2+ Breast Cancer cells in vivo. **(A)** Schematic diagram of the mouse xenograft tumor model. (**B)** Representative images of subcutaneous xenograft tumors from the Vector and STARD10-OE groups. (**C, D)** The volumes and weights of subcutaneous xenografts from the Vector and STARD10-OE groups. (**E)** Representative images of intracellular lipid droplets in tumor tissues from the Vector and STARD10-OE groups. Scale bar = 100 μm. (**F)** H&E staining images of tumor tissues from the Vector and STARD10-OE groups. Scale bar = 200 μm. (**G)** Schematic diagram of the mouse lung metastasis model. (**H)** Representative images of lung metastatic nodules on the surface. (**I)** The metastatic nodules were stained by H&E for histological analysis (left) and the number of metastatic nodules was statistically analyzed and was shown in the bar graph (right). *n* = 4. **P < 0.01.

To evaluate whether STARD10 overexpression promotes tumorigenesis in vivo, we established a murine breast cancer model by subcutaneously implanting STARD10-overexpressing SKBR3 cells (Figure 4A). Measurement of tumor volume and weight revealed that STARD10 overexpression significantly promoted tumor growth in vivo (Figure 4B−D). Moreover, Oil Red O staining revealed higher lipid droplets accumulation in tumor tissues from the STARD10-overexpressing group than in those from the control group (Figure 4E). The pathological features of the xenografts were examined by H&E staining of tissue sections (Figure 4F). No significant difference in body weight was observed between the two groups of mice throughout the experiment (*Supplementary Fig. S11*), suggesting that STARD10 acts specifically on tumor cells without obvious effects on the overall physiological status of the mice.

We next investigated the effect of STARD10 on breast cancer metastasis using a lung colonization model, in which STARD10-overexpressing SKBR3 cells were injected into mice via the tail vein (Figure 4G). As shown in Figure 4H, STARD10 overexpression significantly increased the metastatic colonization of SKBR3 cells in the lungs. Consistent with this, H&E staining of lung sections revealed a significant increase in the number of lung metastatic nodules in the STARD10-overexpressing group compared with the control group (Figure 4I).

Given the small sample size (*n* = 4 per group), we further validated the robustness of these findings using Mann‑Whitney U test, permutation test, and post‑hoc power analysis. All supplementary analyzes consistently supported the statistical significance of the observed differences, with detailed results provided in *Supplementary Table 2*.

### 2.5. STARD10 activates the cAMP/PKA/CREB1 signaling pathway in HER2+ breast cancer

**Figure 5.**
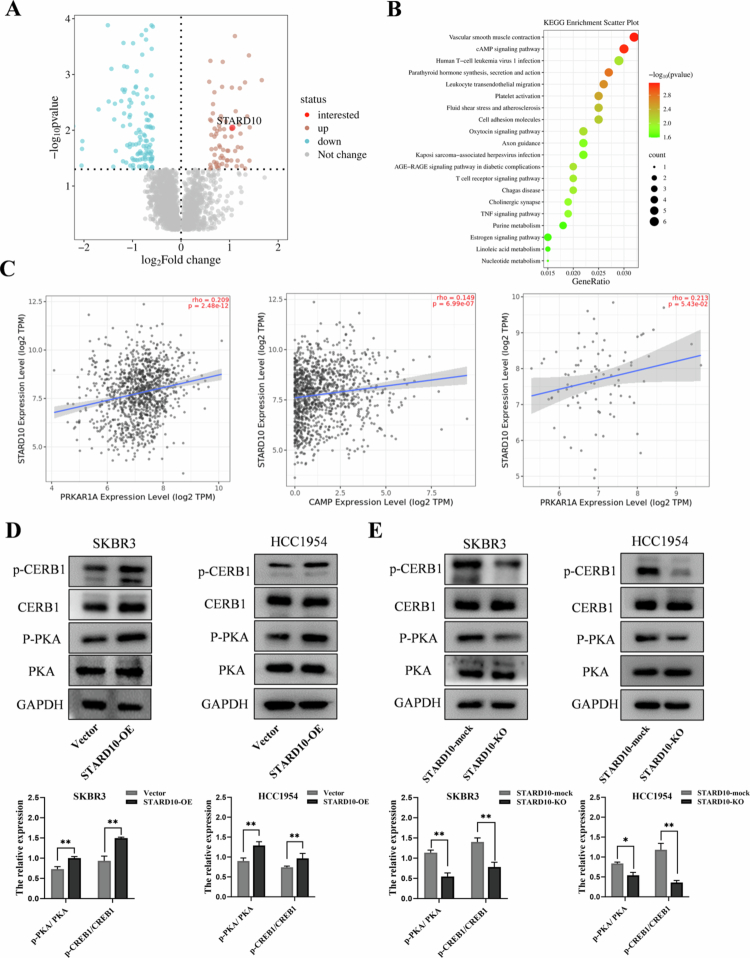
STARD10 activates the cAMP/PKA/CREB1 signaling pathway in HER2+ breast cancer. **(A)** Volcano plot comparing STARD10 overexpression and control SKBR3 cells. **(B)** KEGG pathway analysis of RNA-seq from STARD10 overexpression and control SKBR3 cells. **(C)** Correlation analysis between STARD10 expression and the levels of PRKAR1A, cAMP using TIMER2.0. (D) Western blot analysis of *p*-CREB1, CREB1, *p*-PKA, and PKA expression levels in STARD10-overexpressing SKBR3 and HCC1954 cells compared to control cells. *n* = 3. *P < 0.05, **P < 0.01. (E) Western blot analysis of *p*-CREB1, CREB1, *p*-PKA, and PKA expression levels in STARD10-knockout SKBR3 and HCC1954 cells compared to control cells. *n* = 3. *P < 0.05, **P < 0.01.

To explore the mechanism by which STARD10 regulates malignant phenotypes and lipid droplets metabolism in HER2+ breast cancer, RNA-seq analysis was performed on control and STARD10-overexpressing SKBR3 cells. The RNA-seq results revealed 202 differentially expressed genes (DEGs) (Q value < 0.05, |log_2_FoldChange| >1) in STARD10-overexpressing cells, including 78 upregulated genes and 124 downregulated genes (Figure 5A). To reveal the potential functions of these DEGs, we performed Kyoto Encyclopedia of Genes and Genomes (KEGG) enrichment analyzes. KEGG enrichment analysis revealed that these DEGs were significantly enriched in the cAMP/PKA signaling pathway (Figure 5B), which has been previously implicated in lipid droplets formation.^24^ ^,^ ^25^

Further analysis of TCGA data showed that STARD10 mRNA expression positively correlated with the expression of cAMP/PKA/CREB1 pathway components (PRKAR1A and cAMP) in a pan-breast cancer cohort (R = 0.209 and 0.149, respectively). A significant positive correlation between STARD10 and PRKAR1A expression was also observed specifically in the HER2+ subtype (R = 0.213) (Figure 5C). To further validate this association at single‑cell resolution, we analyzed in‑house single‑cell RNA‑seq data from breast cancer samples encompassing multiple subtypes.^26^ We focused on tumor epithelial subclusters with high ERBB2 expression (representing HER2‑enriched cells). As shown in *Supplementary Fig. S12*, in ERBB2‑high tumor epithelial subclusters (e.g., subclusters 4 and 8), STARD10, PRKAR1A and CREB1 were concomitantly upregulated, whereas subclusters (e.g., subclusters 3, 6, and 11) with high STARD10 but low ERBB2 did not show such co‑expression. This supports a HER2‑dependent correlation between STARD10 and the PKA/CREB1 pathway. The association was validated at the protein level by Western blotting. The results demonstrated that STARD10 overexpression significantly upregulated the expression levels of *p*-PKA and *p*-CREB1 (Figure 5D), whereas STARD10 knockout markedly downregulated the expression of these two phosphorylated proteins (Figure 5E). Moreover, high levels of cAMP (FALL-39) and CREB1 expression predicted poorer clinical outcomes in breast cancer patients (*Supplementary Fig. S13*). Collectively, these results suggest that STARD10 may activate the cAMP/PKA/CREB1 pathway in HER2+ breast cancer.

### 2.6. The cAMP/PKA/CREB1 signaling axis mediates STARD10-driven malignant phenotypes and lipid droplets accumulation

**Figure 6.**
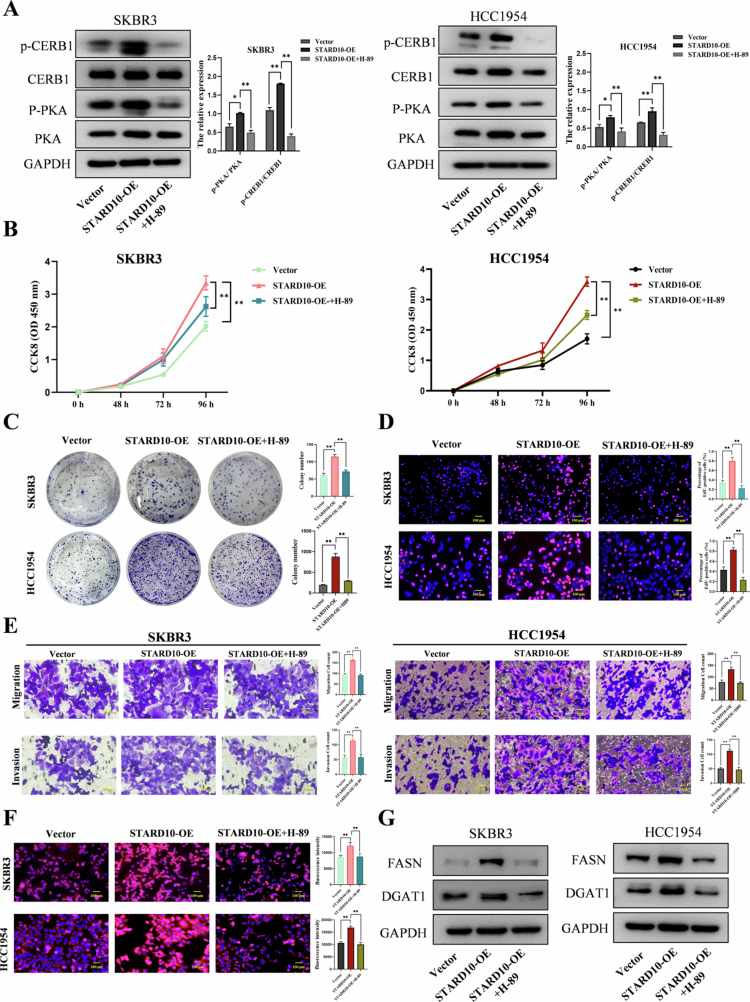
The cAMP/PKA/CREB1 signaling axis mediates STARD10-driven malignant phenotypes and lipid droplets accumulation. **(A)** Western blot was performed to evaluate the expression of *p*-CREB1, CREB1, *p*-PKA, and PKA in SKBR3 and HCC1954 cell lines treated with or without H-89. **(B–D)** CCK-8, colony formation and EdU assays were performed to evaluate the proliferation capacity of SKBR3 and HCC1954 cells treated with or without H-89. Scale bar = 100 μm. **(E)** Transwell assays were performed to evaluate migration and invasion capabilities of SKBR3 and HCC1954 cells treated with or without H-89. Scale bar = 50 μm. **(F)** Representative images of intracellular lipid droplets in SKBR3 and HCC1954 cells stained with LD540 following treatment with H-89. The corresponding quantification shows the fluorescence intensity of lipid droplets. Scale bar = 100 μm. **(G)** Western blot was performed to evaluate the expression of FASN and DGAT1 in SKBR3 and HCC1954 cell lines treated with or without H-89. *n* = 3. *P < 0.05, **P < 0.01.

To verify whether the cAMP/PKA/CREB1 signaling axis mediates the regulatory effects of STARD10 in HER2+ breast cancer cells, we conducted a rescue experiment using H-89, a highly specific inhibitor targeting the cAMP/PKA signaling pathway, at a concentration of 40 μmol/L. Western blot analysis was performed to detect the expression levels of key proteins in the pathway, and the results confirmed that H-89 treatment at 40 μmol/L effectively inhibited the STARD10-induced upregulation of *p*-PKA and *p*-CREB1 expression (Figure 6A and B), thereby blocking the activation of the cAMP/PKA/CREB1 signaling axis.

Subsequent functional assays, including CCK-8, colony formation, EdU, Transwell, and 3D spheroid formation assays, demonstrated that 40 μmol/L H-89 treatment significantly reversed the promoting effects of STARD10 on the proliferation, migration, invasion, and stemness of HER2+ breast cancer cells (Figure 6B−E, *Supplementary Fig. S14*). Furthermore, LD540 and Nile Red staining and Western blotting showed that H‑89 treatment at the same concentration also reduced the accumulation of intracellular lipid droplets induced by STARD10 overexpression (Figure 6F and G; *Supplementary Figs. S15* and *S16*). Collectively, these findings demonstrate that the cAMP/PKA/CREB1 signaling axis serves as a key mediator through which STARD10 regulates the malignant phenotypes and abnormal lipid metabolism of HER2+ breast cancer cells.

## 3. Discussion

HER2+ breast cancer remains a formidable clinical challenge due to its high invasiveness and strong propensity for recurrence and metastasis.^27^ ^,^ ^28^ Recent advances in tumor metabolism have highlighted the pivotal role of lipid metabolic reprogramming in tumorigenesis and progression,^29^ with the crosstalk between lipid transport and intracellular signaling emerging as a key regulator of malignant phenotypes.^30^ ^,^ ^31^ The STARD protein family mediates intracellular lipid transfer and participates in diverse pathophysiological processes.^32^ ^,^ ^33^ However, the specific role of its member STARD10 in cancer, especially in HER2+ breast cancer, remains poorly understood, and the underlying mechanisms are yet to be elucidated. This study elucidated the biological function and molecular mechanisms of STARD10 in lipid transport and malignant progression in HER2+ breast cancer, thereby providing new therapeutic targets and strategies for this subtype.

Previous studies have predominantly focused on the physiological functions of STARD10, such as maintaining lipid homeostasis in pancreatic *β*-cells.^15^ ^,^ ^34^ ^,^ ^35^ The functions of STARD10 in oncology are poorly defined, with only limited associations with cell proliferation reported.^36^ Our study demonstrates that STARD10 not only promotes the proliferation, migration, and invasion of HER2+ breast cancer cells in vitro, but also enhances tumor growth and lung metastasis in mouse models. Extending beyond the previous narrow focus on proliferation, this study clarifies the critical role of STARD10 in driving metastasis, underscoring its multifaceted functions during tumor progression.

A key finding is that STARD10 overexpression activates the cAMP/PKA/CREB1 signaling axis. Previous studies have indicated that the cAMP/PKA pathway exerts context-dependent tumor-suppressive or oncogenic effects.^37^ ^,^ ^38^ For instance, this pathway has been identified as a core oncogenic driver in certain liver cancer models,^39^ ^,^ ^40^ whereas its activation inhibits tumor progression in glioblastoma.^41^ Here, we demonstrate for the first time a regulatory link between STARD10 and the cAMP/PKA/CREB1 pathway in HER2+ breast cancer. Inhibition of the cAMP/PKA/CREB1 pathway effectively reversed the enhanced cell proliferation, migration, and invasion mediated by STARD10 overexpression. Consistent with this, analysis of the TCGA database showed a positive correlation between STARD10 expression and key components of this pathway. These results collectively demonstrate that STARD10 drives the malignant progression of HER2+ breast cancer by activating the cAMP/PKA/CREB1 signaling axis.

Lipid droplets are central organelles for dynamic lipid storage and redistribution, and their status reflects cellular lipid metabolic activity.^42^ ^,^ ^43^ Given that lipid droplet status directly reflects cellular lipid metabolic activity, we examined changes in lipid droplets to assess the impact of STARD10 on lipid transport. We found that STARD10 overexpression significantly increased lipid droplets formation, whereas its knockout reduced lipid droplets formation. Furthermore, prior evidence suggests the cAMP/PKA/CREB1 signaling axis can regulate lipid droplets metabolism in MCF-7 cells.^24^ The present study extends this finding to HER2+ breast cancer cells, demonstrating that inhibition of the cAMP/PKA/CREB1 pathway significantly reverses STARD10 overexpression ‑induced lipid droplets accumulation and the increased expression of FASN and DGAT1. FASN and DGAT1 are key enzymes in lipid droplets synthesis and directly determine the rate and extent of lipid droplets formation.^44^ ^,^ ^45^ Based on the above results, this study demonstrates that STARD10 overexpression promotes lipid droplets formation by activating the cAMP/PKA/CREB1 pathway to upregulate key lipogenic enzymes.

Notably, in addition to the cAMP/PKA/CREB1 axis, other signaling cascades including the PI3K/AKT/mTOR pathway is implicated in the pathogenesis of hyperproliferative diseases.^46^ Meanwhile, the NF-κB and p38 MAPKα pathways play critical regulatory roles in inflammation and metabolic reprogramming.^47^ Collectively, these observations suggest that multiple signaling pathways converge during pathological processes and synergistically contribute to tumor progression.^48-50^ Whether STARD10 interacts with these pathways warrants further investigation.

This study has several limitations. First, although we analyzed the expression of STARD10 using public databases and clinical cohorts, we have not performed immunohistochemical staining in HER2+ breast cancer tissues, nor have we examined the co-expression of STARD10, HER2 and key molecules of the cAMP/PKA/CREB1 signaling pathway in clinical samples. Immunohistochemical validation using HER2+ tissue microarrays is an essential supplement to this study, and we will complete this work in our future research. On this basis, we will also conduct large-scale tissue staining experiments to clarify the correlations among the above indicators and their relationships with the clinical outcomes of patients. Second, the number of mice per group in the animal experiments was relatively small (*n* = 4 per group). Although we validated the robustness of the results using multiple non‑parametric and permutation tests, the limited sample size still partially restricts the translational potential of our findings. Moreover, the therapeutic effect of H‑89 was not validated in vivo. Therefore, to maintain the rigor of our conclusions, future studies should increase the sample size to at least six mice per group and perform in vivo H‑89 administration experiments to further validate the role of the cAMP/PKA/CREB1 axis in the tumor microenvironment. Third, although this study confirmed that STARD10 can activate the PKA/CREB1 pathway, its upstream mechanism (especially whether STARD10 mediates this effect by regulating the synthesis or secretion of cAMP) remains unclear, and the causal link between STARD10 and the cAMP/PKA/CREB1 axis is currently mainly supported by H-89 treatment. In the future, we will systematically dissect the molecular association between STARD10 and cAMP homeostasis and PKA activation, and conduct CREB1 genetic rescue experiments to confirm the causal relationship of this pathway, thereby improving the regulatory model proposed in this study. Fourth, although this study confirmed that STARD10 positively regulates key lipogenic enzymes and promotes lipid droplet formation, it focused only on lipid synthesis and lipid droplet metabolism. The regulatory effect of STARD10 on lipid oxidation remains unexplored, particularly regarding whether STARD10 modulates mitochondrial fatty acid transport mediated by the CPT family. In future studies, functional experiments such as measurement of fatty acid oxidation rate and CPT enzyme activity, combined with metabolomic analysis, will be performed to comprehensively elucidate the regulatory pattern and molecular function of STARD10 in overall lipid metabolic flux.

In summary, our research revealed that STARD10 is significantly upregulated in HER2+ breast cancer and associated with unfavorable patient outcomes. Furthermore, we identified a novel role for STARD10 in activating cAMP/PKA/CREB1 signaling axis to regulate tumor progression and lipid metabolism. These findings not only provide novel insights into the molecular mechanisms underlying HER2+ breast cancer but also identify STARD10 as a potential prognostic biomarker and therapeutic target. Targeting STARD10 or its downstream the cAMP/PKA/CREB1 signaling axis represents a promising direction for future therapy. Future studies are warranted to validate the prognostic value of STARD10 in larger clinical cohorts and to evaluate its translational potential as a therapeutic target using additional preclinical in vivo models.

## 4. Materials and methods

### 4.1. Cell culture and reagents

The human breast cancer cell lines SKBR3 (SCSP-5243, Chinese Academy of Sciences Cell Bank) and HCC1954 (TCHu245) were cultured in RPMI 1640 (31800022, Gibco), the human embryonic kidney epithelial cell line 293 T (SCSP-502) and the normal mammary epithelial cell line MCF-10A (SCSP-575) were cultured in DMEM/F12 (12500062, Gibco). All culture media were supplemented with 10% fetal bovine serum (A5256701, ExCell Bio) and 1% penicillin-streptomycin (15140122, Beyotime). These cells were incubated in a humidified incubator at 37 °C with 5% CO_2_. Detailed cell information and specifications of all reagents and equipment are listed in *Supplementary File 1 (Tabs. S1, S5, S6)*.

### 4.2. Lentivirus construction and cell transfection

The STARD10 gene sequence was cloned into the TK-PCDH-copGFP-T2A-Puro lentiviral vector (Tsingke Biotechnology). The plasmid and Lipofectamine 3000 (L3000015, ThermoFisher) were mixed with fresh serum-free, antibiotic-free medium, and the mixture was added dropwise to 293 T cells for viral packaging. The viral solution was concentrated, purified, and used to infect the cancer cells. After 48 h, the cancer cells were stably transduced with viral particles, and cells were subsequently treated with puromycin (ST551, Beyotime) to eliminate uninfected cells. Finally, the expression efficiency of STARD10 was detected by Western blot and RT-qPCR.

### 4.3. CRISPR/Cas9 knockout

To target the CDS region of the STARD10 gene, highly specific sgRNA sequences were selected using the online platform CRISPR Design Tool and chemically synthesized by Sangon Biotech. The sgRNA sequences were cloned into PX459 vector downstream of the U6 promoter using the BbsI restriction enzyme (ER1011, ThermoFisher). Lipofectamine 3000 transfection reagent was used to transfect the recombinant plasmid. After 48 h of transfection, the optimal working concentration of puromycin was determined through gradient selection to establish stable knockout cell lines. Finally, the knockout efficiency was assessed by Western blot and RT-qPCR.

### 4.4. CCK-8 assay

Cells were seeded in 96-well plates at a density of 2000 cells per well. Subsequently, 10% CCK-8 reagent (FC101-01, TransGen) was added to each well at 0, 48, 72, and 96 h. After the plates were incubated at 37 °C for 2 h, the OD value was measured using a multi-functional microplate reader (BioTek Epoch2 Instruments).

### 4.5. EdU-594 cell proliferation assay

The cells were cultured overnight in 12-well plates in preparation for the EdU incorporation experiment. Next, the cells were incubated with medium containing EdU working solution (C0078S, Beyotime) at the concentration of 10 μmol/L at 37 °C for 2 h. Then, the cells were fixed with 4% paraformaldehyde (P0099, Beyotime) and permeabilized with 0.3% Triton X-100 solution (ST1723, Beyotime). Following permeabilization, the Click Additive Solution was applied, and the cell nuclei were stained with Hoechst 33342 (C1022, Beyotime). EdU-positive cells were quantified using a fluorescence microscope (Olympus).

### 4.6. Colony formation assay

The cells were seeded in 6-well plates at a density of 1500 cells per well and cultured for 14 days. The culture supernatant was then removed, and the cells were fixed with 4% paraformaldehyde. Then the fixed cells were stained with crystal violet solution (Y268091, Beyotime), and images were acquired using an inverted microscope.

### 4.7. 3D culture assay

The cells were suspended in RPMI 1640 medium containing 0.25% (w/v) 4000cp methylcellulose (ST1510, Beyotime), then cell suspension was inoculated onto the lid of the petri dish at 1.0 × 10^3^ cells/40 µL, with PBS added to the dish. The cell spheroids were observed and photographed using an inverted fluorescence microscope after 14 days.

### 4.8. Transwell assay

For the migration assay, 400 µL of serum-free culture medium containing 4 × 10^4^ cells was added to the upper chamber of Transwell apparatus with an 8 µm membrane (FTW043-48Ins, Beyotime). The lower chamber was filled with 400 µL of culture medium supplemented with 20% FBS. For the invasion assay, the upper chamber membrane was coated with Matrigel (C0372, Beyotime). After incubation at 37 °C for 48 h, the cells that had migrated through or invaded the membrane were fixed with 4% paraformaldehyde and stained with crystal violet solution. Images were then acquired using an inverted microscope.

### 4.9. LD540 staining

LD540 staining was used to detect and visualize the lipid droplets content in the cells. The cells were seeded on circular coverslips in 12-well plates and black 96-well plates. After 48 h, the cells were washed twice with PBS and fixed with fixation buffer for 30 min. Then, the cells were incubated with LD540 staining solution (C2050S, Beyotime) in the dark for 20 min. After staining, the cells were washed with PBS, and stained with Hoechst 33342 for 10 min. Images were obtained by scanning the LD540-stained cell smears using a fluorescence microscope and fluorescence intensity was measured using a multi-functional microplate reader (BioTek Epoch2).

### 4.10. Nile red staining

Lipid droplets were detected using a Nile Red kit (C2051S, Beyotime) according to the manufacturer’s instructions. HER2+ cells were seeded onto circular coverslips in 12-well plates and in black 96-well plates. After 48 h, cells were washed twice with PBS, fixed for 30 min, and stained with Nile Red for 20 min. Cells were then counterstained with Hoechst 33342 for 10 min to label nuclei, washed three times with PBS, and imaged under a fluorescence microscope. Fluorescence intensity was quantified using a multifunctional microplate reader.

### 4.11. Animal models

Female BALB/c-nu mice (3-4 weeks old, 12-15g; BALB/cNj-Foxn1nu/Gpt, Huachuang Sino) were housed in pathogen-free facilities with individual ventilation, constant humidity (40%-60%) and temperature (23 ± 1℃), ad libitum access to food and water, and a regular 12 h light/12 h dark cycle. At 5 weeks of age, mice were randomly assigned to two groups (*n* = 4 per group). A suspension of control cells (Vector) and experimental cells (STARD10-OE) in PBS were injected into the subcutaneous area of the axilla of each mouse at a dose of 5 × 10^5^ cells/100 µL. The volume of the transplanted tumors was measured every 2 days via Vernier calipers. The tumor volume was calculated via the following formula: volume (mm^3^) = 0.5 × L (mm) × S^2^ (mm2). Body weight was recorded every 4 days to monitor general health of mice. Next, the nude mice were euthanized by cervical dislocation after 7 weeks, and the tumor tissues were excised and weighed. For the lung metastasis assay, a total of 2 × 10^6^ cells/100 µL were injected into the tail vein of nude mice. After 10 weeks, the nude mice were euthanized by cervical dislocation, and lung tissues were collected and processed for H&E staining. All animal procedures were approved by the Animal Ethics Committee of Northwest University (No. NWU-AWC-20240910M) and complied with the ARRIVE guidelines.

### 4.12. Western blot

Total cellular proteins were extracted with RIPA lysate buffer (P0013B, Beyotime) and protein concentration was determined using a BCA protein concentration assay kit (PC0020, Solarbio). The protein samples were separated on SDS-polyacrylamide gels and transferred onto PVDF membranes (IPVH00010, Merck). After being blocked with 10% skim milk (P0216, Beyotime) at room temperature for 2 h, the membranes were incubated with the appropriate primary antibodies at 4 °C overnight. The primary antibodies utilized included STARD10, PKA, *p*-PKA, CREB1, *p*-CREB1, GAPDH, *β*-Actin, DGAT1, and FASN. HRP-conjugated secondary antibodies were subsequently incubated for 2 h at room temperature. Bands were visualized using ECL reagents (34580, ThermoFisher) and imaged with a ChemiDoc™ MP Imaging System (LAS-4000, Fujifilm). All antibodies used are listed in *Supplementary File 1 (Tabs. S2, S3)*.

### 4.13. RT-qPCR

Total RNA was extracted from cells using TransZol Up reagent (R0016, TransGen) and cDNA was synthesized by reverse transcription using EasyScript® (AE101-02, TransGen). RT-qPCR was performed using PerfectStart® Green qPCR SuperMix (AQ602-01, TransGen) according to the manufacturer's instructions. The results were normalized to the expression of the control gene (GAPDH) using the 2^-ΔΔCt^ method. All primer sequences can be found in *Supplementary File 1 (Tab. S4)*.

### 4.14. RNA-sequencing and bioinformatics analysis

Total RNA was extracted from SKBR3 cells in the control group and the STARD10 overexpression group using TransZol Up reagent. RNA sequencing was performed by Shanghai Biotechnology Corporation. DEGs were identified with a threshold of fold change >1 and q value < 0.05. KEGG pathway enrichment analysis was subsequently conducted for these DEGs.

### 4.15. Analyzes based on public datasets

STARD10 expression in breast cancer and normal tissues was analyzed using the GEPIA2 tool based on the TCGA database. The prognostic value of STARD10 in breast cancer was evaluated by Kaplan-Meier Plotter. Correlation analysis was performed using TIMER 2.0. STARD10 expression in representative HER2+ breast cancer cell lines were obtained from the Human Protein Atlas database. Clinical information for the HER2+ breast cancer cohort from the GEO database is listed in *Supplementary Table 1*. All data acquisition and usage complied with relevant database policies.

### 4.16. Statistical analysis

All experiments were independently repeated at least three times, and data are shown as mean ± standard deviation. Statistical analyzes were conducted using GraphPad Prism 8.0. Student’s t-test was used for two-group comparisons, while one-way or two-way ANOVA was applied for multiple-group analyzes. Considering the limited sample size (*n* = 4 per group) of in vivo experiments, we further validated the statistical robustness via Mann-Whitney U test (exact, two-sided) and permutation tests (10,000 permutations). Post-hoc power analysis based on Cohen’s d effect size was also performed, with detailed results provided in the Supplementary Materials. Statistical significance was defined as *P < 0.05, **P < 0.01, and ns represents no significant difference.

## Supplementary Material

Supplementary File 1.docx Supplementary File 1.docx

Supplementary Table 1.docx Supplementary Table 1.docx

Supplementary Table 2.docx Supplementary Table 2.docx

Supplementary Figures.docx Supplementary Figures.docx

Supplementary Material ARRIVE Guidelines Checklist

## Data Availability

The raw sequencing data supporting the results of this article have been deposited in the NCBI Sequence Read Archive (SRA) under the BioProject accession PRJNA1455363. A temporary reviewer access link is provided at: https://dataview.ncbi.nlm.nih.gov/object/PRJNA1455363?reviewer=4g7i54cognccqkk21mbne10jnf. The dataset will be released to the public upon acceptance of the manuscript for publication.
