# Supplementary material for: STARD10 promotes progression of HER2+ breast cancer and intracellular lipid metabolism via the cAMP/PKA/CREB1 signaling axis
Source: Cancer Biol Ther. 2026 Jun 15;27(1):2688544. doi: 10.1080/15384047.2026.2688544 (PMC13274131; doi:10.1080/15384047.2026.2688544)
Supplement: Supplementary File 1.docx [file KCBT_A_2688544_SM7931.docx]

**Tab.S1 Cell Lines**

| **Cell Lines Name** | **Cat. No** | **Company** |
| --- | --- | --- |
| SKBR3 | SCSP-5243 | Chinese Academy of Sciences Cell Bank |
| HCC1954 | TCHu245 | Chinese Academy of Sciences Cell Bank |
| 293T | SCSP-502 | Chinese Academy of Sciences Cell Bank |
| MCF-10A | SCSP-575 | Chinese Academy of Sciences Cell Bank |

**Tab.S2 Primary antibody information**

| **Antibody Name** | **kDa** | **Dilution multiple** | **Source** | **Company** | **Cat. No** |
| --- | --- | --- | --- | --- | --- |
| STARD10 | 37 | 1:1000 | Rabbit | Proteintech | 17048-1-AP |
| PKA | 42 | 1:5000 | Mouse | Proteintech | 67491-1-Ig |
| p-PKA | 42 | 1:500 | Rabbit | Affinity | AF7246 |
| CREB1 | 42 | 1:2000 | Rabbit | Proteintech | 12208-1-AP |
| p-CREB1 | 42 | 1:1000 | Rabbit | Proteintech | 28792-1-AP |
| GAPDH | 35 | 1:50000 | Mouse | Proteintech | 60004-1-Ig |
| β-Actin | 42 | 1:2000 | Rabbit | Proteintech | 20536-1-AP |
| DGAT1 | 54 | 1:1000 | Mouse | UpingBio | YP-mAb-08717 |
| FASN | 273 | 1:500 | Mouse | UpingBio | YP-mAb-02636 |

**Tab.S3** **Secondary antibody information**

| **Antibody Name** | **Dilution multiple** | **Company** | **Cat. No** |
| --- | --- | --- | --- |
| HRP-Goat Anti-Rabbit | 1:2000 | Proteintech | RGAR001 |
| HRP-Goat Anti-Mouse | 1:2000 | Proteintech | RGAM001 |

**Tab.S4** **Primer information**

| **GENE** | **Forward primer (5’-3’)** | **Reverse primer (5’-3’)** |
| --- | --- | --- |
| STARD10 | CTGTGGAGATGGATCGGACG | GGTAAGGAGCCTTTGGGGGTC |
| GAPDH | GGAGTCCACTGGCGTCTTCA | GTCATGAGTCCTTCCACGATACC |

**Tab.S5 Reagents and Drug Information**

| **Reagents** | **Usage** | **Company** | **Cat. No** |
| --- | --- | --- | --- |
| RPMI 1640 | Cell culture medium | Gibco | 31800022 |
| DMEM/F12 | Cell culture medium | Gibco | 12500062 |
| Fetal Bovine Serum | 10% | ExCell Bio | A5256701 |
| Penicillin-streptomycin | 1% | ThermoFisher | 15140122 |
| Lipofectamine 3000 | Plasmid transfection reagent | ThermoFisher | L3000015 |
| Puromycin | Screening stable cell lines | Beyotime | ST551 |
| BbsI restriction enzyme | Vector enzyme digestion | ThermoFisher | ER1011 |
| CCK-8 | 10% | TransGen | FC101-01 |
| EdU 594 | 10 μmol/L | Beyotime | C0078S |
| Paraformaldehyde | 4% | Beyotime | P0099 |
| TritonX-100 | 0.3% | Beyotime | ST1723 |
| Hoechst 33342 | 1% | Beyotime | C1022 |
| Crystal violet solution | 0.1% | Beyotime | Y268091 |
| 4000cp methylcellulose | 0.25% (w/v) | Beyotime | ST1510 |
| Matrigel | 1:8 dilution | Beyotime | C0372 |
| LD540 staining solution | Lipid droplet staining | Beyotime | C2050S |
| Nile Red Staining solution | Lipid droplet staining | Beyotime | C2051S |
| RIPA lysate buffer | Total protein extraction | Beyotime | P0013B |
| EasyScript® Reverse Transcriptase | cDNA synthesis | TransGen | AE101-02 |
| TransZol Up | Total RNA extraction | Beyotime | R0016 |
| PerfectStart® Green qPCR SuperMix | RT-qPCR analysis | TransGen | AQ602-01 |
| BCA Protein Assay Kit | Protein quantification | Solarbio | PC0020 |
| PVDF membranes | Western blot transfer | Merck | IPVH00010 |
| Skim milk | 10% | Beyotime | P0216 |
| ECL reagents | Western blot visualization | ThermoFisher | 34580 |
| Hematoxylin and Eosin Staining | Tissue histologic staining | Beyotime | C0105S |
| H-89 inhibitor | 40 μmol/L | Beyotime | S1643 |

**Tab.S6 Main Equipment**

| **Equipment Name** | **Company** | **Cat. No** |
| --- | --- | --- |
| 60mm petri dish | ThermoFisher | 150462 |
| 100mm petri dish | ThermoFisher | 150466 |
| 6-well plate | ThermoFisher | 140675 |
| 12-well plate | ThermoFisher | 150628 |
| 24-well plate | ThermoFisher | 142475 |
| 96-well plate | ThermoFisher | 260887 |
| Black 96-well plate | ThermoFisher | 437112 |
| BeyoGold™ Transwell | Beyotime | FTW043-48Ins |
| CO2 Incubator | ThermoFisher | 360 |
| Cellometer Mini Cell Counter | Nexcelom | Mini-006-0635 |
| Multi-functional microplate reader | BioTek Epoch2 | 1906284 |
| Fluorescence Microscope | Olympus | IX73 |
| Inverted Microscope | Olympus | CKX31 |
| Centrifuge | ThermoFisher | Fresco 21 |
| PCR Machine | ThermoFisher | A51685 |
| Electrophoresis Unit | Bio-Rad | 1658033 |
| ChemiDoc™ MP Imaging System | Fujifilm | LAS-4000 |
