# Supplementary material for: STARD10 promotes progression of HER2+ breast cancer and intracellular lipid metabolism via the cAMP/PKA/CREB1 signaling axis
Source: Cancer Biol Ther. 2026 Jun 15;27(1):2688544. doi: 10.1080/15384047.2026.2688544 (PMC13274131; doi:10.1080/15384047.2026.2688544)
Supplement: Supplementary Table 1.docx [file KCBT_A_2688544_SM7928.docx]

**Clinical data of 75 patients with HER2+ breast cancer from the GEO cohort.**

| **ID** | **sample** | **STARD10** | **gender** | **TUMOR SIZE** | **pathologic_N** | **pathologic_M** | **pathologic_stage** | **PR_Status** | **ER status** |
| --- | --- | --- | --- | --- | --- | --- | --- | --- | --- |
| TCGA-A8-A08H-01 | Age≥50（n=58） | 0.35475 | FEMALE | T2 | N0 | M0 | Stage II | PR-positive（n=45） | ER-positive（n=57） |
| TCGA-AO-A12D-01 | Age＜50（n=21） | 0.078125 | FEMALE | T1 | N1 | M0 | Stage II | PR-negative（n=34） | ER-Negative（n=20） |
| TCGA-A8-A08X-01 | Age＜50（n=21） | 2.7395 | FEMALE | T4 | N3 | M0 | Stage III | PR-negative（n=34） | PR-positive（n=45） |
| TCGA-C8-A1HF-01 | Age＜50（n=21） | 0.443125 | FEMALE | T2 | N0 | M0 | Stage II | PR-positive（n=45） | ER-Negative（n=20） |
| TCGA-E2-A14Y-01 | Age＜50（n=21） | 0.59175 | FEMALE | T2 | N0 | M0 | Stage II | PR-positive（n=45） | ER-positive（n=57） |
| TCGA-C8-A12L-01 | Age≥50（n=58） | 0.59925 | FEMALE | T2 | N0 | M0 | Stage II | PR-negative（n=34） | ER-Negative（n=20） |
| TCGA-BH-A0B7-01 | Age＜50（n=21） | 0.6 | FEMALE | T2 | N1 | M0 | Stage II | PR-positive（n=45） | ER-positive（n=57） |
| TCGA-A8-A097-01 | Age≥50（n=58） | 0.66175 | FEMALE | T2 | N1 | M0 | Stage II | PR-positive（n=45） | ER-positive（n=57） |
| TCGA-BH-A0DD-01 | Age≥50（n=58） | 0.663875 | MALE | T2 | N1 | M0 | Stage II | PR-positive（n=45） | ER-positive（n=57） |
| TCGA-C8-A135-01 | Age≥50（n=58） | 0.779875 | FEMALE | T2 | N1 | M0 | Stage II | PR-negative（n=34） | ER-Negative（n=20） |
| TCGA-A2-A0CX-01 | Age≥50（n=58） | 0.96475 | FEMALE | T2 | N0 | M0 | Stage II | PR-negative（n=34） | ER-positive（n=57） |
| TCGA-A8-A08S-01 | Age≥50（n=58） | 1.049875 | FEMALE | T1 | N1 | M0 | Stage II | PR-positive（n=45） | ER-positive（n=57） |
| TCGA-E2-A14W-01 | Age≥50（n=58） | 1.062375 | MALE | T2 | N0 | M0 | Stage II | PR-positive（n=45） | ER-positive（n=57） |
| TCGA-AN-A0FT-01 | Age≥50（n=58） | 1.086125 | FEMALE | T2 | N1 | M0 | Stage II | PR-positive（n=45） | ER-positive（n=57） |
| TCGA-A2-A0EY-01 | Age≥50（n=58） | 1.1095 | FEMALE | T2 | N1 | M0 | Stage II | PR-negative（n=34） | ER-positive（n=57） |
| TCGA-C8-A12Z-01 | Age＜50（n=21） | 1.11125 | FEMALE | T2 | N1 | M0 | Stage II | PR-negative（n=34） | ER-Negative（n=20） |
| TCGA-B6-A0RH-01 | Age≥50（n=58） | 1.1595 | FEMALE | T2 | N0 | M0 | Stage II | PR-positive（n=45） | ER-positive（n=57） |
| TCGA-C8-A132-01 | Age≥50（n=58） | 1.174 | FEMALE | T2 | N1 | M0 | Stage II | PR-positive（n=45） | ER-positive（n=57） |
| TCGA-AN-A04C-01 | Age≥50（n=58） | 1.2305 | FEMALE | T2 | N1 | M0 | Stage II | PR-negative（n=34） | ER-Negative（n=20） |
| TCGA-A2-A0D1-01 | Age≥50（n=58） | 1.298625 | FEMALE | T2 | N0 | M0 | Stage II | PR-negative（n=34） | ER-Negative（n=20） |
| TCGA-AN-A0FD-01 | Age≥50（n=58） | 1.3295 | FEMALE | T2 | N0 | M0 | Stage II | PR-positive（n=45） | ER-positive（n=57） |
| TCGA-A2-A0YG-01 | Age≥50（n=58） | 1.3975 | FEMALE | T2 | N3 | M0 | Stage III | PR-positive（n=45） | ER-positive（n=57） |
| TCGA-A8-A099-01 | Age≥50（n=58） | 1.416 | FEMALE | T4 | N3 | MX | Stage II | PR-positive（n=45） | ER-positive（n=57） |
| TCGA-AR-A1AX-01 | Age≥50（n=58） | 1.53675 | FEMALE | T1 | N0 | M0 | Stage I | PR-positive（n=45） | ER-positive（n=57） |
| TCGA-A8-A06X-01 | Age≥50（n=58） | 1.559875 | FEMALE | T3 | N0 | M0 | Stage II | PR-negative（n=34） | ER-positive（n=57） |
| TCGA-A8-A09I-01 | Age≥50（n=58） | 1.5685 | FEMALE | T2 | N0 | M0 | Stage II | PR-positive（n=45） | ER-positive（n=57） |
| TCGA-AR-A1AT-01 | Age≥50（n=58） | 1.569375 | FEMALE | T2 | N0 | M0 | Stage II | PR-positive（n=45） | ER-positive（n=57） |
| TCGA-A8-A0A7-01 | Age≥50（n=58） | 1.58925 | FEMALE | T2 | N1 | M0 | Stage II | PR-negative（n=34） | ER-Negative（n=20） |
| TCGA-A8-A08P-01 | Age≥50（n=58） | 1.643125 | FEMALE | T2 | N2 | M0 | Stage III | PR-positive（n=45） | ER-positive（n=57） |
| TCGA-A2-A0T1-01 | Age≥50（n=58） | 1.64475 | FEMALE | T3 | N3 | M0 | Stage III | PR-negative（n=34） | ER-Negative（n=20） |
| TCGA-C8-A12Q-01 | Age≥50（n=58） | 1.674375 | FEMALE | T1 | N2 | M0 | Stage III | PR-negative（n=34） | ER-Negative（n=20） |
| TCGA-C8-A138-01 | Age≥50（n=58） | 1.6805 | FEMALE | T2 | N2 | M0 | Stage III | PR-negative（n=34） | ER-positive（n=57） |
| TCGA-BH-A18U-01 | Age≥50（n=58） | 1.752 | FEMALE | T2 | N2 | M0 | Stage III | PR-positive（n=45） | ER-positive（n=57） |
| TCGA-BH-A0B4-01 | Age≥50（n=58） | 1.754875 | MALE | T2 | N2 | M0 | Stage III | PR-positive（n=45） | ER-positive（n=57） |
| TCGA-BH-A18M-01 | Age＜50（n=21） | 1.762375 | FEMALE | T3 | N1 | M0 | Stage III | PR-positive（n=45） | ER-positive（n=57） |
| TCGA-BH-A0EE-01 | Age≥50（n=58） | 1.817125 | FEMALE | T3 | N0 | M0 | Stage II | PR-negative（n=34） | ER-Negative（n=20） |
| TCGA-AN-A0AJ-01 | Age≥50（n=58） | 1.82425 | FEMALE | T3 | N0 | M0 | Stage II | PR-positive（n=45） | ER-positive（n=57） |
| TCGA-A2-A04W-01 | Age≥50（n=58） | 1.828375 | FEMALE | T2 | N1 | M0 | Stage II | PR-negative（n=34） | ER-Negative（n=20） |
| TCGA-A2-A0SY-01 | Age≥50（n=58） | 1.849875 | FEMALE | T3 | N1 | M0 | Stage III | PR-positive（n=45） | ER-positive（n=57） |
| TCGA-E2-A1B0-01 | Age≥50（n=58） | 1.859 | FEMALE | T2 | N2 | M0 | Stage III | PR-negative（n=34） | ER-Negative（n=20） |
| TCGA-BH-A0C0-01 | Age≥50（n=58） | 1.9585 | FEMALE | T1 | N1 | M0 | Stage II | PR-positive（n=45） | ER-positive（n=57） |
| TCGA-E2-A15E-01 | Age＜50（n=21） | 1.97725 | FEMALE | T1 | N1 | M0 | Stage II | PR-positive（n=45） | ER-positive（n=57） |
| TCGA-A1-A0SM-01 | Age≥50（n=58） | 1.9985 | MALE | T2 | N0 | M0 | Stage II | PR-negative（n=34） | ER-positive（n=57） |
| TCGA-C8-A137-01 | Age＜50（n=21） | 2.058125 | FEMALE | T2 | N1 | M0 | Stage II | PR-negative（n=34） | ER-Negative（n=20） |
| TCGA-A8-A09G-01 | Age≥50（n=58） | 2.089125 | FEMALE | T3 | N3 | M0 | Stage III | PR-negative（n=34） | ER-positive（n=57） |
| TCGA-A8-A08B-01 | Age≥50（n=58） | 2.10925 | FEMALE | T2 | N0 | M0 | Stage II | PR-negative（n=34） | ER-positive（n=57） |
| TCGA-AO-A0JM-01 | Age＜50（n=21） | 2.12125 | FEMALE | T2 | N1 | M0 | Stage II | PR-positive（n=45） | ER-positive（n=57） |
| TCGA-AR-A0TX-01 | Age≥50（n=58） | 2.13775 | FEMALE | T1 | N1 | M0 | Stage II | PR-positive（n=45） | ER-positive（n=57） |
| TCGA-BH-A18R-01 | Age≥50（n=58） | 2.171375 | FEMALE | T2 | N1 | M0 | Stage II | PR-negative（n=34） | ER-positive（n=57） |
| TCGA-E2-A14V-01 | Age≥50（n=58） | 2.1865 | FEMALE | T2 | N1 | M0 | Stage II | PR-positive（n=45） | ER-positive（n=57） |
| TCGA-A8-A09N-01 | Age≥50（n=58） | 2.20075 | FEMALE | T2 | N3 | M0 | Stage III | PR-positive（n=45） | ER-positive（n=57） |
| TCGA-D8-A140-01 | Age≥50（n=58） | 2.24025 | FEMALE | T2 | N1 | M0 | Stage II | PR-positive（n=45） | ER-positive（n=57） |
| TCGA-AO-A0JE-01 | Age≥50（n=58） | 2.289 | FEMALE | T2 | N2 | M0 | Stage III | PR-negative（n=34） | ER-Negative（n=20） |
| TCGA-BH-A0C7-01 | Age＜50（n=21） | 2.343125 | FEMALE | T2 | N1 | M0 | Stage II | PR-negative（n=34） | ER-positive（n=57） |
| TCGA-C8-A12P-01 | Age≥50（n=58） | 2.35075 | FEMALE | T2 | N1 | M0 | Stage II | PR-negative（n=34） | ER-Negative（n=20） |
| TCGA-B6-A0I9-01 | Age≥50（n=58） | 2.398625 | FEMALE | T3 | N2 | M1 | Stage IV | PR-positive（n=45） | ER-positive（n=57） |
| TCGA-E2-A152-01 | Age≥50（n=58） | 2.474 | FEMALE | T1 | N0 | M0 | Stage I | PR-negative（n=34） | ER-positive（n=57） |
| TCGA-C8-A12T-01 | Age＜50（n=21） | 2.52875 | FEMALE | T2 | N0 | M0 | Stage II | PR-positive（n=45） | ER-positive（n=57） |
| TCGA-BH-A0AW-01 | Age≥50（n=58） | 2.531375 | FEMALE | T1 | N1 | M0 | Stage II | PR-negative（n=34） | ER-positive（n=57） |
| TCGA-A8-A076-01 | Age≥50（n=58） | 2.551125 | FEMALE | T2 | N0 | M0 | Stage II | PR-positive（n=45） | ER-positive（n=57） |
| TCGA-A8-A07I-01 | Age≥50（n=58） | 2.563 | FEMALE | T2 | N2 | M0 | Stage III | PR-negative（n=34） | ER-positive（n=57） |
| TCGA-AQ-A04L-01 | Age＜50（n=21） | 2.57575 | FEMALE | T2 | N0 | MX | Stage II | PR-negative（n=34） | ER-positive（n=57） |
| TCGA-E2-A14P-01 | Age≥50（n=58） | 2.632125 | FEMALE | T2 | N3 | M0 | Stage III | PR-negative（n=34） | ER-Negative（n=20） |
| TCGA-A2-A04X-01 | Age＜50（n=21） | 2.68225 | FEMALE | T2 | N0 | M0 | Stage II | PR-positive（n=45） | ER-positive（n=57） |
| TCGA-A2-A0EQ-01 | Age≥50（n=58） | 2.84125 | FEMALE | T2 | N0 | M0 | Stage II | PR-negative（n=34） | ER-Negative（n=20） |
| TCGA-BH-A0DZ-01 | Age＜50（n=21） | 2.851125 | FEMALE | T2 | N1 | M0 | Stage II | PR-positive（n=45） | ER-positive（n=57） |
| TCGA-AO-A12C-01 | Age＜50（n=21） | 2.860625 | FEMALE | T2 | N1 | M0 | Stage II | PR-positive（n=45） | ER-positive（n=57） |
| TCGA-C8-A1HL-01 | Age＜50（n=21） | 2.899375 | FEMALE | T3 | N1 | M0 | Stage III | PR-negative（n=34） | ER-positive（n=57） |
| TCGA-A8-A08G-01 | Age＜50（n=21） | 3.006 | FEMALE | T2 | N0 | M0 | Stage II | PR-positive（n=45） | ER-positive（n=57） |
| TCGA-AR-A0TQ-01 | Age＜50（n=21） | 3.1115 | FEMALE | T3 | N1 | M0 | Stage III | PR-negative（n=34） | ER-positive（n=57） |
| TCGA-AQ-A04H-01 | Age≥50（n=58） | 3.23075 | FEMALE | T2 | N2 | MX | Stage III | PR-positive（n=45） | ER-positive（n=57） |
| TCGA-A8-A07B-01 | Age≥50（n=58） | 3.250125 | FEMALE | T2 | N0 | M0 | Stage II | PR-positive（n=45） | ER-positive（n=57） |
| TCGA-E2-A1B1-01 | Age＜50（n=21） | 3.565875 | FEMALE | T2 | N1 | M0 | Stage II | PR-positive（n=45） | ER-positive（n=57） |
| TCGA-E2-A15H-01 | Age＜50（n=21） | 3.644125 | FEMALE | T1 | N1 | M0 | Stage II | PR-positive（n=45） | ER-positive（n=57） |
| TCGA-A8-A07P-01 | Age≥50（n=58） | 3.7265 | FEMALE | T2 | N1 | M0 | Stage II | PR-positive（n=45） | ER-positive（n=57） |
