# Supplementary material for: STARD10 promotes progression of HER2+ breast cancer and intracellular lipid metabolism via the cAMP/PKA/CREB1 signaling axis
Source: Cancer Biol Ther. 2026 Jun 15;27(1):2688544. doi: 10.1080/15384047.2026.2688544 (PMC13274131; doi:10.1080/15384047.2026.2688544)
Supplement: Supplementary Table 2.docx [file KCBT_A_2688544_SM7929.docx]

Supplementary Tables S1-S4 present the raw in vivo data and the results of multiple statistical analyses used to validate the robustness of the findings despite the small sample size (n = 4 per group). These include the original t‑test, Mann‑Whitney U test, permutation test, and post‑hoc power analysis.

Supplementary Table S1:

Individual in vivo data of tumor volume, tumor weight, and lung metastasis count in Vector and STARD10‑OE groups

| Endpoint | Vector group (n=4) | STARD10‑OE group (n=4) |
| --- | --- | --- |
| Tumor volume (mm³, day 50) | 292.5 | 817.96 |
|  | 91 | 479.23 |
|  | 178.36 | 563.16 |
|  | 187.2 | 611.59 |
| Tumor weight (g) | 0.096 | 0.2767 |
|  | 0.1268 | 0.3333 |
|  | 0.0446 | 0.5059 |
|  | 0.0771 | 0.4366 |
| Lung metastasis  count | 2 | 6 |
|  | 0 | 8 |
|  | 1 | 10 |
|  | 0 | 6 |

Legend: Individual measurements of tumor volume (mm³, day 50), tumor weight (g), and lung metastasis count for each mouse in the Vector (control) and STARD10‑OE groups. n = 4 mice per group.

Supplementary Table S2:

Two‑sample Student’s t‑test results (assuming equal variance) for each in vivo endpoint

| Endpoint | t value | Degrees of freedom | Two‑tailed P value |
| --- | --- | --- | --- |
| Tumor volume (day 50) | 5.187 | 6 | 0.0021 |
| Tumor weight | 5.576 | 6 | 0.0014 |
| Lung metastasis count | 6.306 | 6 | 0.00073 |

Legend: Conventional two‑sample Student’s t‑test (equal variance) comparing Vector and STARD10‑OE groups for each endpoint. Degrees of freedom (df) = 6 for all comparisons.

Supplementary Table S3:

Mann‑Whitney U test (exact, two‑sided) and permutation test (10,000 random permutations) results for each in vivo endpoint

| Endpoint | Mann-Whitney U | Exact two-sided P value | Permutation test P value (10,000 permutations) |
| --- | --- | --- | --- |
| Tumor volume (day 50) | 0 | 0.0286 | < 0.0001 |
| Tumor weight | 0 | 0.0286 | < 0.0001 |
| Lung metastasis count | 0 | 0.0286 | < 0.0001 |

Legend: Non‑parametric Mann‑Whitney U test (exact, two‑sided) and permutation test (10,000 random permutations) results. A Mann‑Whitney U statistic of 0 indicates perfect separation between the two groups. Exact two‑sided P = 0.0286 for all endpoints. Empirical P values from permutation tests are all <0.0001.

Supplementary Table S4:

Post‑hoc power analysis results for each in vivo endpoint based on observed effect sizes (n = 4 per group)

| Endpoint | Cohen's d | Observed power (α=0.05) | Required n for 80% power |
| --- | --- | --- | --- |
| Tumor volume (day 50) | 3.67 | >99% | 3 |
| Tumor weight | 3.94 | >99% | 2 |
| Lung metastasis count | 4.46 | >99.9% | 2 |

Legend: Post‑hoc power analysis based on observed effect sizes (Cohen’s d). α = 0.05. Observed power >99% for all endpoints. The required sample size per group to achieve 80% power is also shown, ranging from 2 to 3 mice.
