# Supplementary material for: STARD10 promotes progression of HER2+ breast cancer and intracellular lipid metabolism via the cAMP/PKA/CREB1 signaling axis
Source: Cancer Biol Ther. 2026 Jun 15;27(1):2688544. doi: 10.1080/15384047.2026.2688544 (PMC13274131; doi:10.1080/15384047.2026.2688544)
Supplement: Supplementary Figures.docx [file KCBT_A_2688544_SM7930.docx]

***Fig.S1* STARD10 expression in various cancer types analyzed using the TIMER2 database**


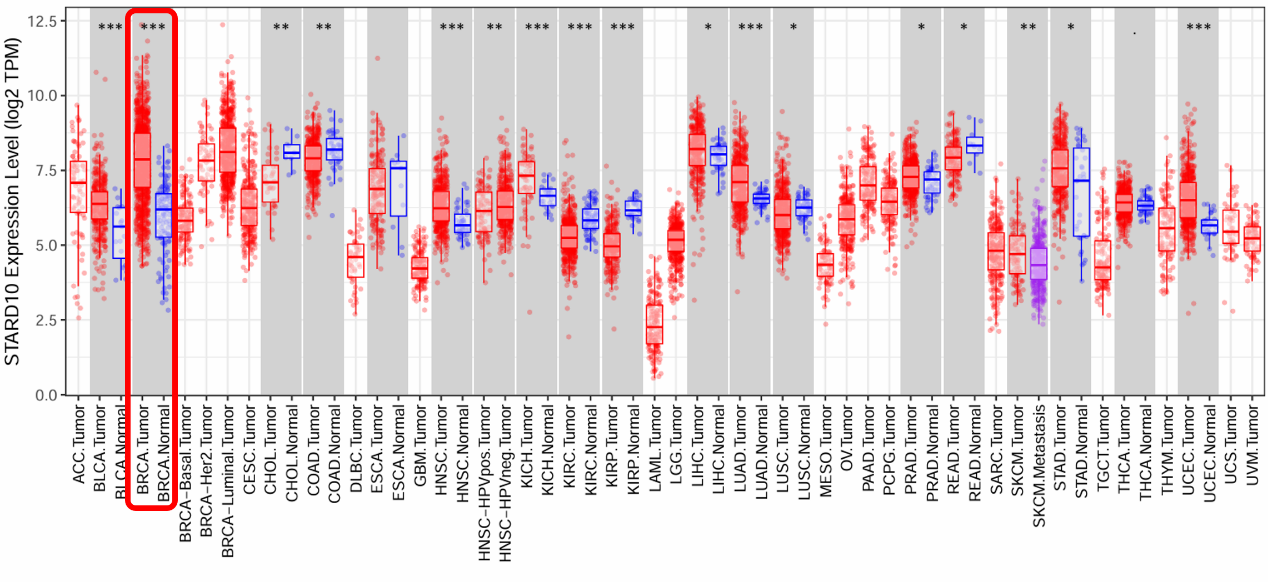


mRNA expression of STARD10 in normal breast tissue and breast cancer tissue was analyzed using the TIMER2 database.

***Fig.S2* Western blot analysis of STARD10 and HER2 expression levels.**

**
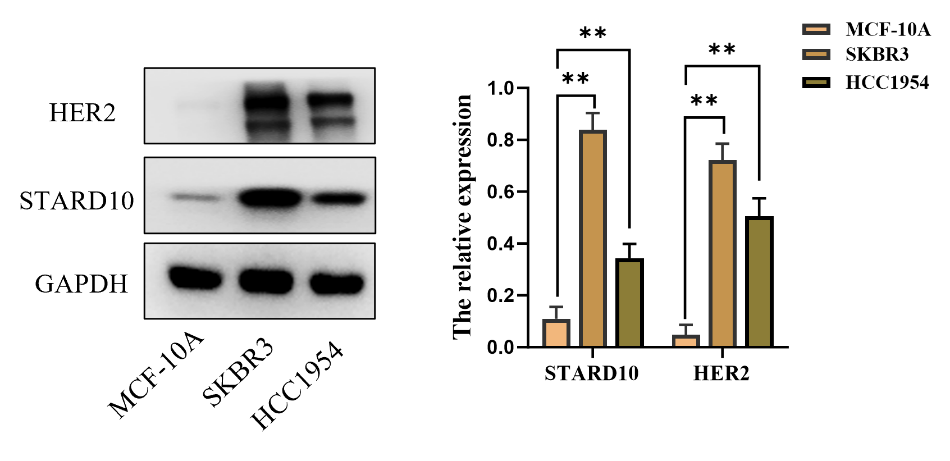
**

Western blot analysis of STARD10 and HER2 expression levels in the normal mammary epithelial cell line MCF-10A and the HER2+ breast cancer cell lines SKBR3 and HCC1954. n = 3. ***P* < 0.01.

***Fig.S3* STARD10 expression in different breast cancer cell lines via the Human Protein Atlas database**


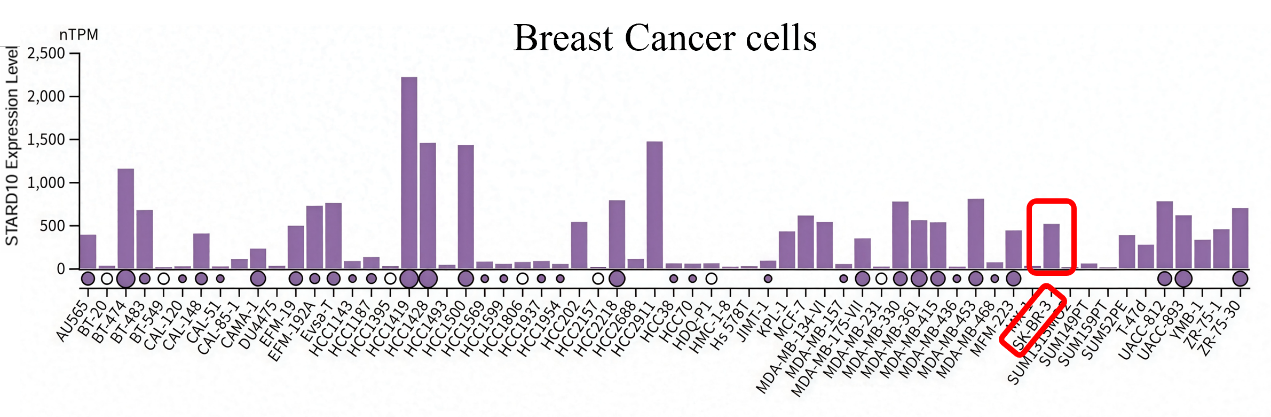


mRNA expression levels of STARD10 in the representative HER2+ breast cancer cell line SKBR3 were analyzed using the Human Protein Atlas database.

***Fig.S4* Lentiviral transduction efficiency in STARD10-overexpressing cell lines.**


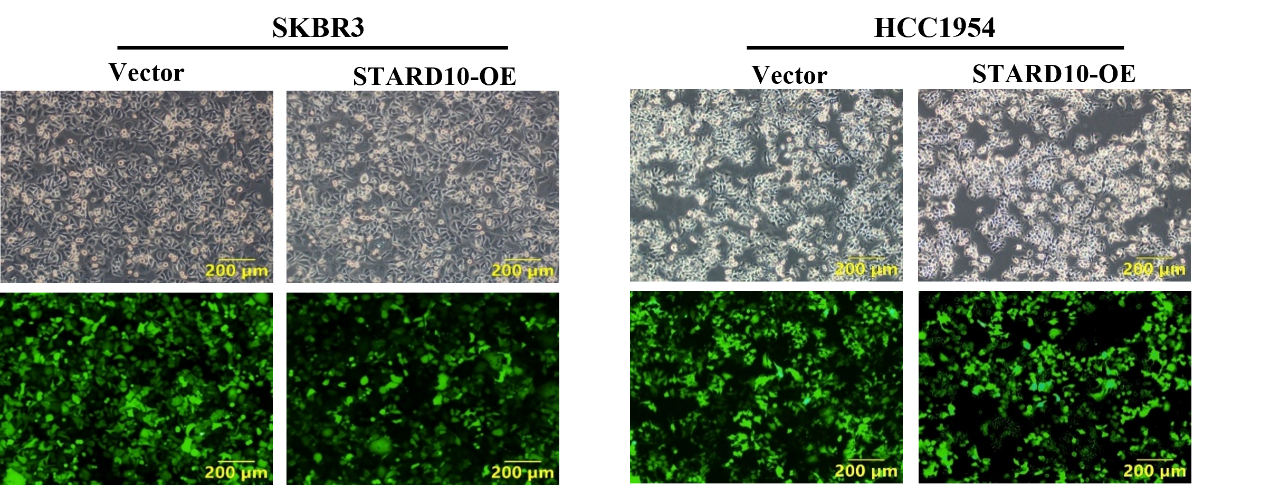


Representative bright-field and GFP fluorescence images of SKBR3 and HCC1954 cells transduced with the empty vector or the STARD10 plasmid are presented. The high GFP positivity rate confirms successful viral transduction. Scale bar = 200 μm. n = 3.

***Fig.S5*** **Effect of STARD10 overexpression on 3D spheroid formation.**


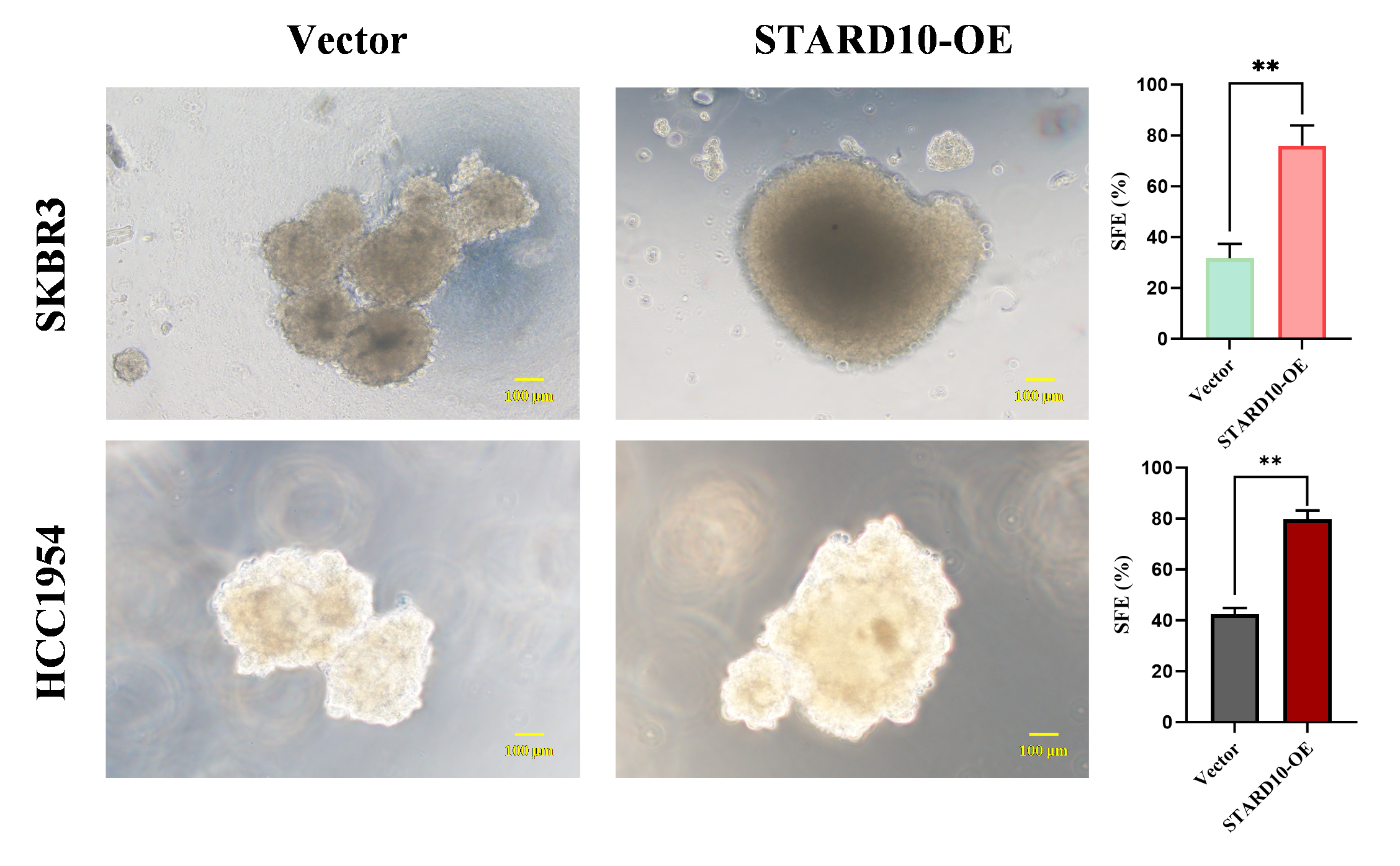


The sphere-forming ability of stable STARD10-overexpressing SKBR3 and HCC1954 cells was assessed by 3D spheroid formation assay. Scale bar = 100 μm. n = 3. ***P* < 0.01.

***Fig.S6* Effect of STARD10 overexpression on lipid droplets formation.**

**
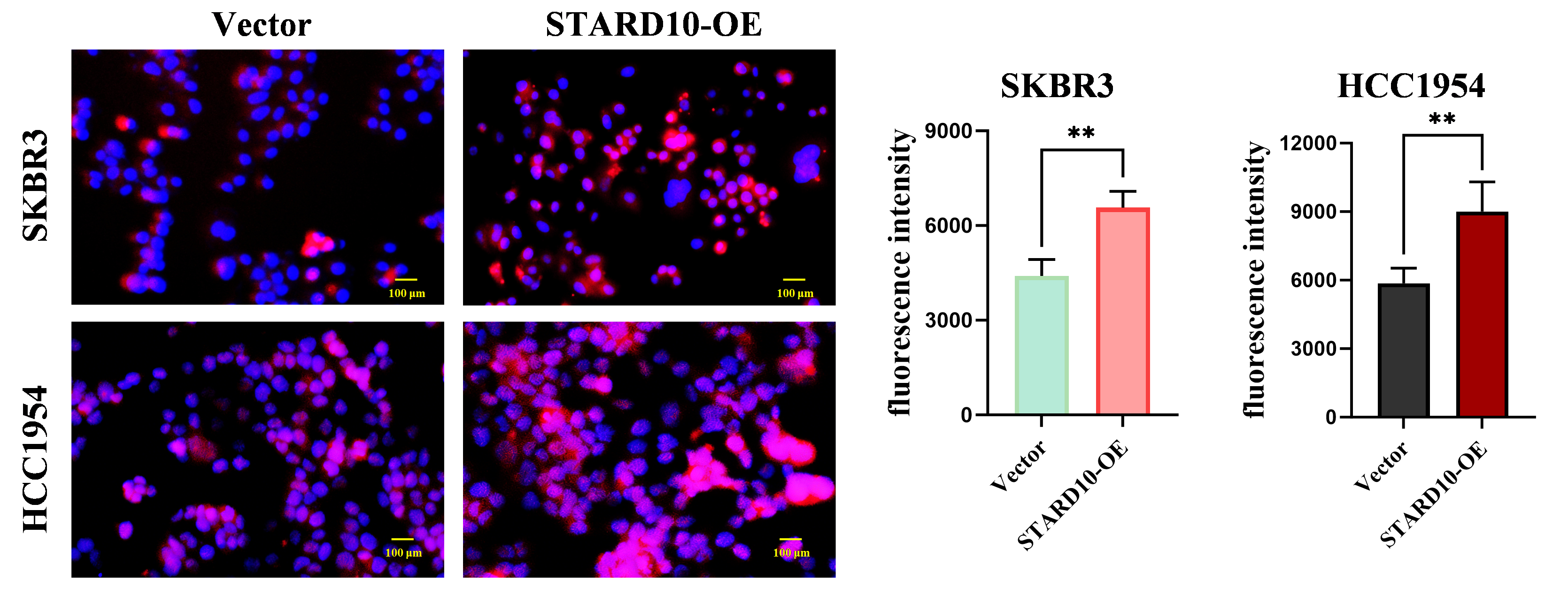
**

Representative images of intracellular lipid droplets in stable STARD10-overexpressing SKBR3 and HCC1954 cells stained with Nile Red staining. The corresponding quantification shows the fluorescence intensity of lipid droplets. Scale bar = 100 μm. n = 3. ***P* < 0.01.

***Fig.S7* Western blot analysis of FASN and DGAT1 expression levels following STARD10 overexpression.**

Statistical analysis of the effects of STARD10-overexpressing SKBR3 and HCC1954 cells on FASN and DGAT1 protein expression. n = 3. ***P* < 0.01.

***Fig.S8* Effect of STARD10 knockout on lipid droplets formation.**


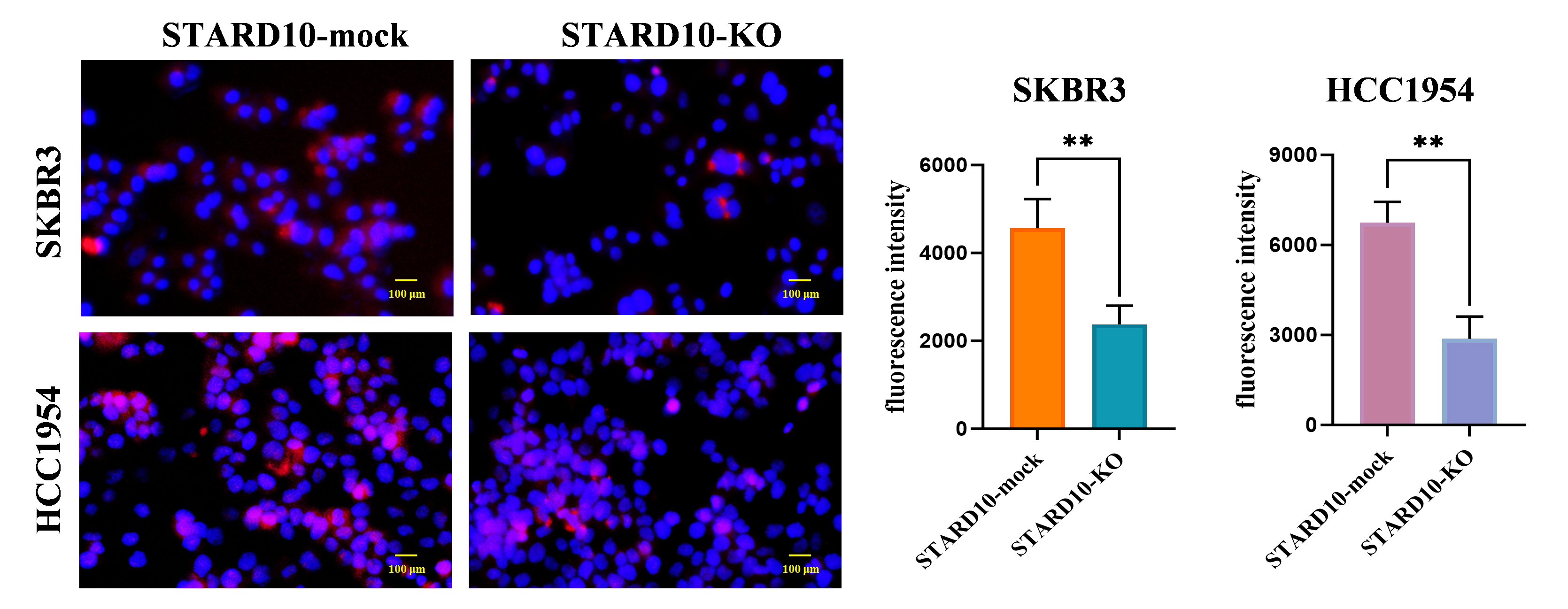


Representative images of intracellular lipid droplets in stable STARD10-knockout SKBR3 and HCC1954 cells stained with Nile Red staining. The corresponding quantification shows the fluorescence intensity of lipid droplets. Scale bar = 100 μm. n = 3. ***P* < 0.01.

***Fig.S9*** **Western blot analysis of FASN and DGAT1 expression levels following STARD10-knockout.**

Statistical analysis of the effects of STARD10-knockout SKBR3 and HCC1954 cells on FASN and DGAT1 protein expression. n = 3. **P* < 0.05, ***P* < 0.01.

***Fig.S10* Effect of STARD10 knockout on 3D spheroid formation.**


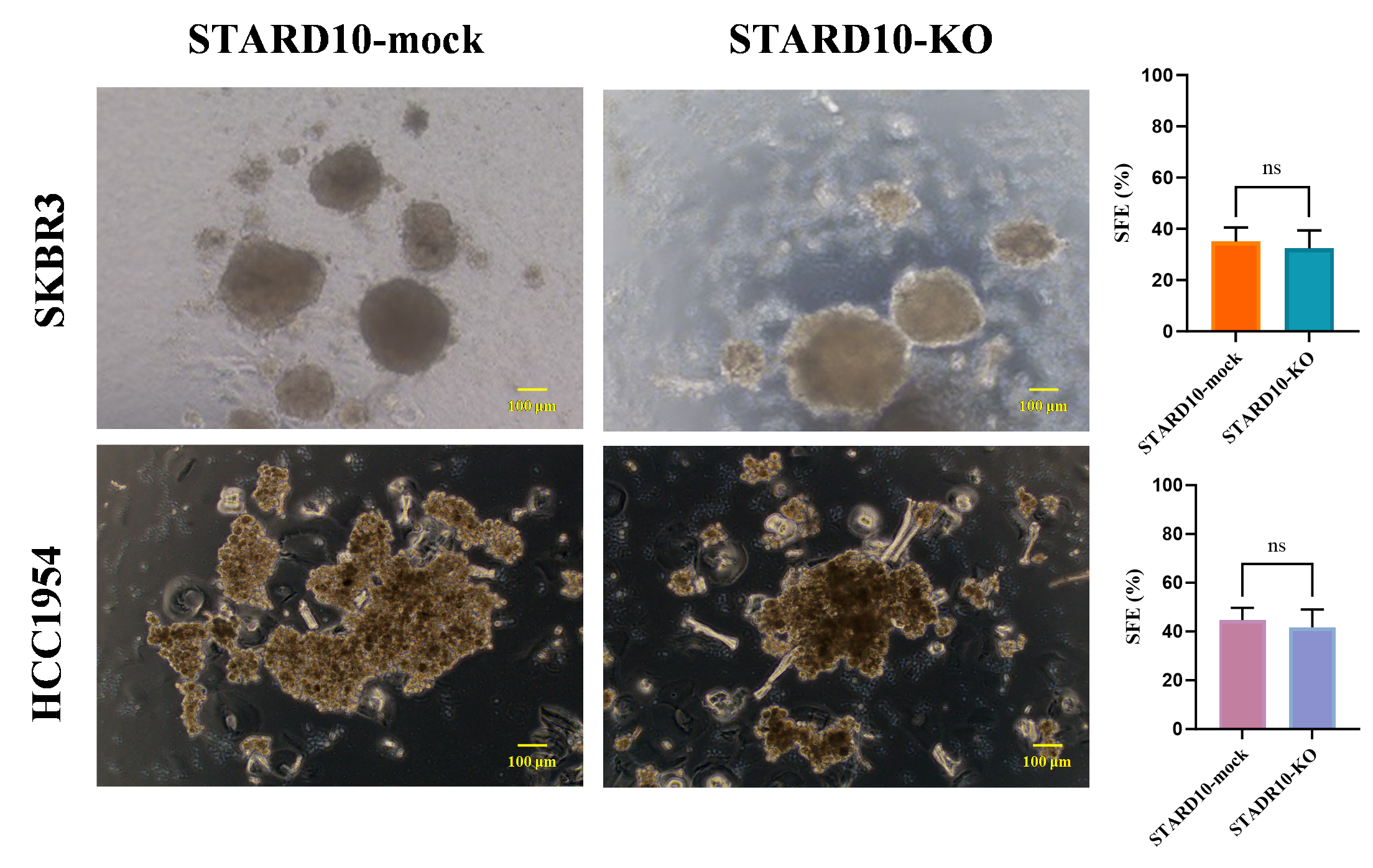


The sphere-forming ability of SKBR3 and HCC1954 cells with STARD10-knockout was assessed using a 3D spheroid formation assay. Scale bar = 100 μm. n = 3. ns, not significant.

***Fig.S11 Monitoring of body weight in mice in the Vector group and the STARD10-OE group*.**


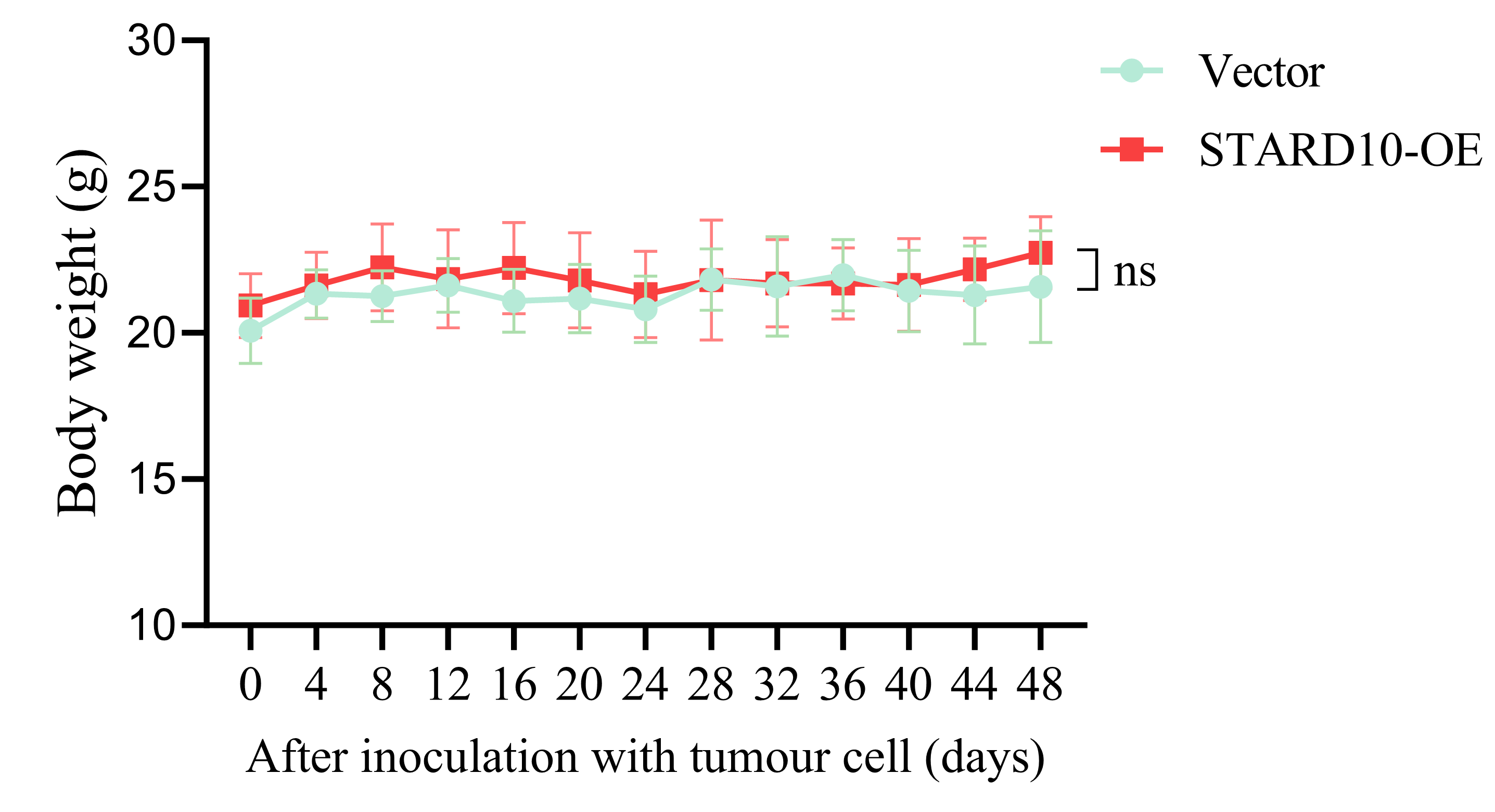


Body weight of mice in the Vector and STARD10-OE groups was measured every 4 days after tumor cell injection. No significant difference in body weight was observed between the two groups of mice throughout the experiment. n = 4. ns, not significant.

***Fig.S12 Single‑cell transcriptomic landscape of tumor epithelial subclusters and expression of ERBB2, STARD10, and PRKAR1A in breast cancer.***


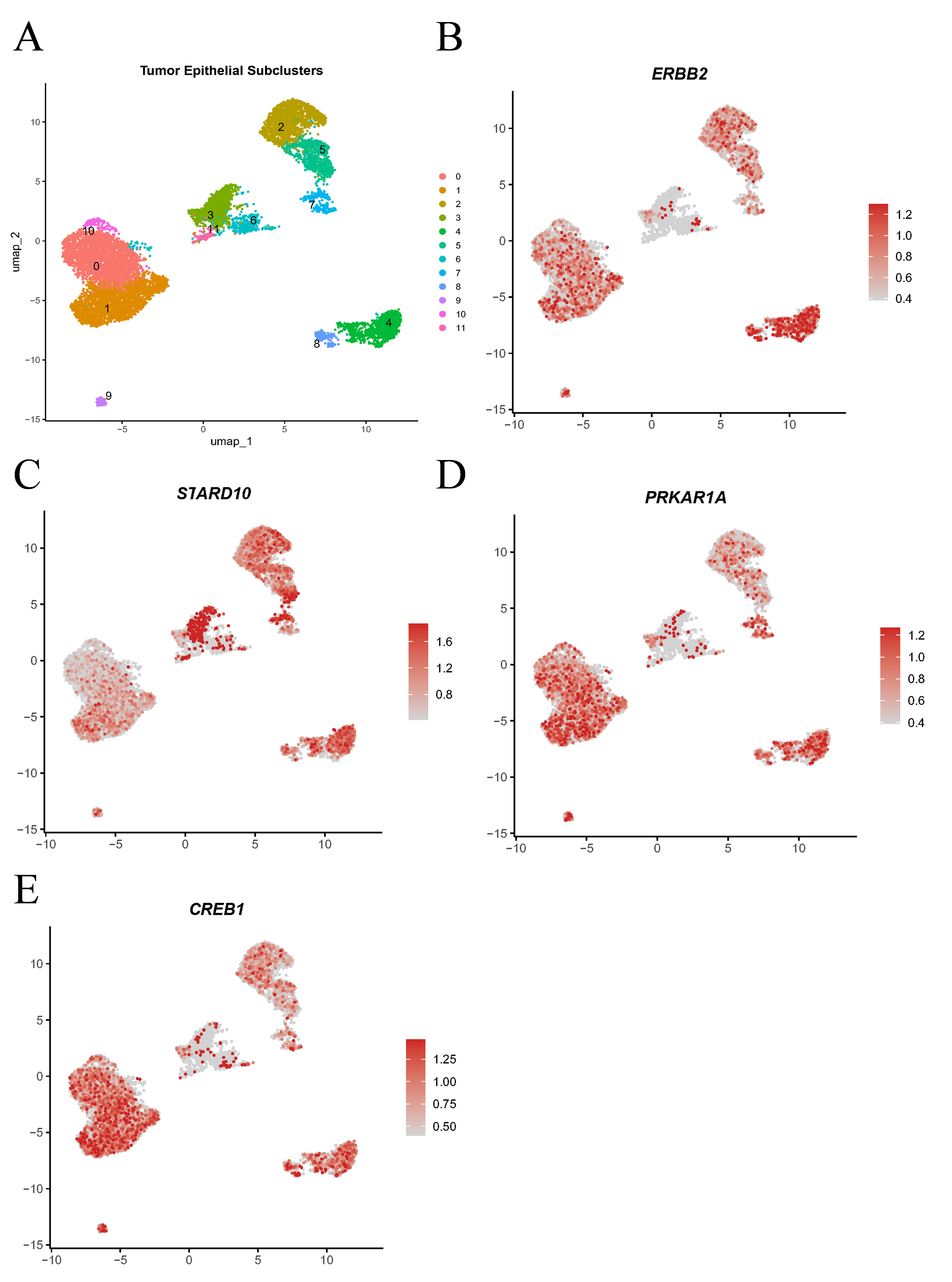


(A) UMAP plot showing the clustering of tumor epithelial subclusters identified from single‑cell RNA‑seq data of clinical breast cancer samples. Each number (0-11) represents a distinct tumor epithelial subcluster. (B) UMAP plot showing the expression level of ERBB2 (encoding HER2) across the tumor epithelial subclusters. Color scale indicates normalized expression intensity. (C) UMAP plot showing the expression level of STARD10 across the tumor epithelial subclusters. (D) UMAP plot showing the expression level of PRKAR1A (a key component of the PKA pathway) across the tumor epithelial subclusters. (E) UMAP plot showing the expression level of CREB1 (a key component of the PKA pathway) across the tumor epithelial subclusters.

***Fig.S13* Clinical prognosis analysis of cAMP and CREB1.**


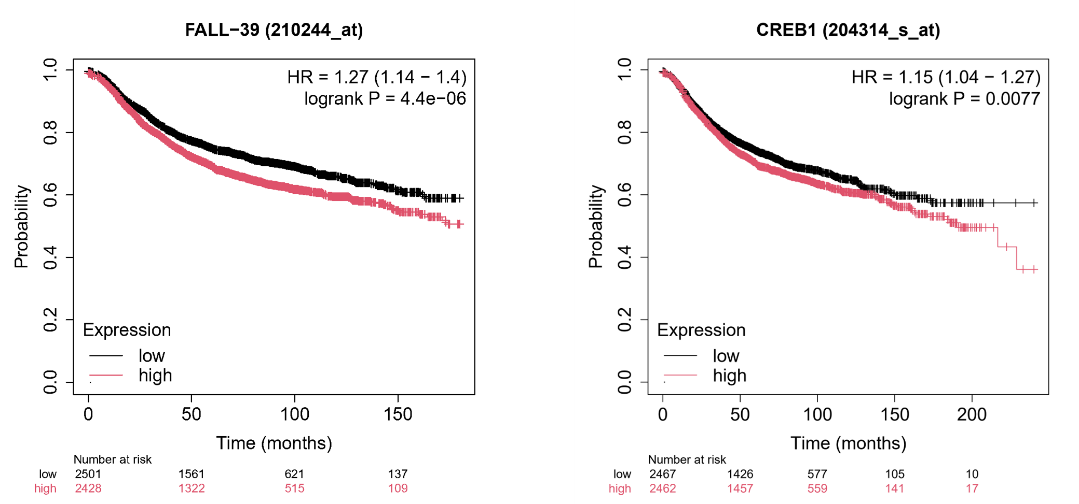


Relapse-free survival (RFS) curves of breast cancer patients with low versus high cAMP and CREB1 expression in the TCGA cohort.

***Fig.S14* Effect of H-89 inhibitor on 3D spheroid formation in STARD10-overexpressing cells.**


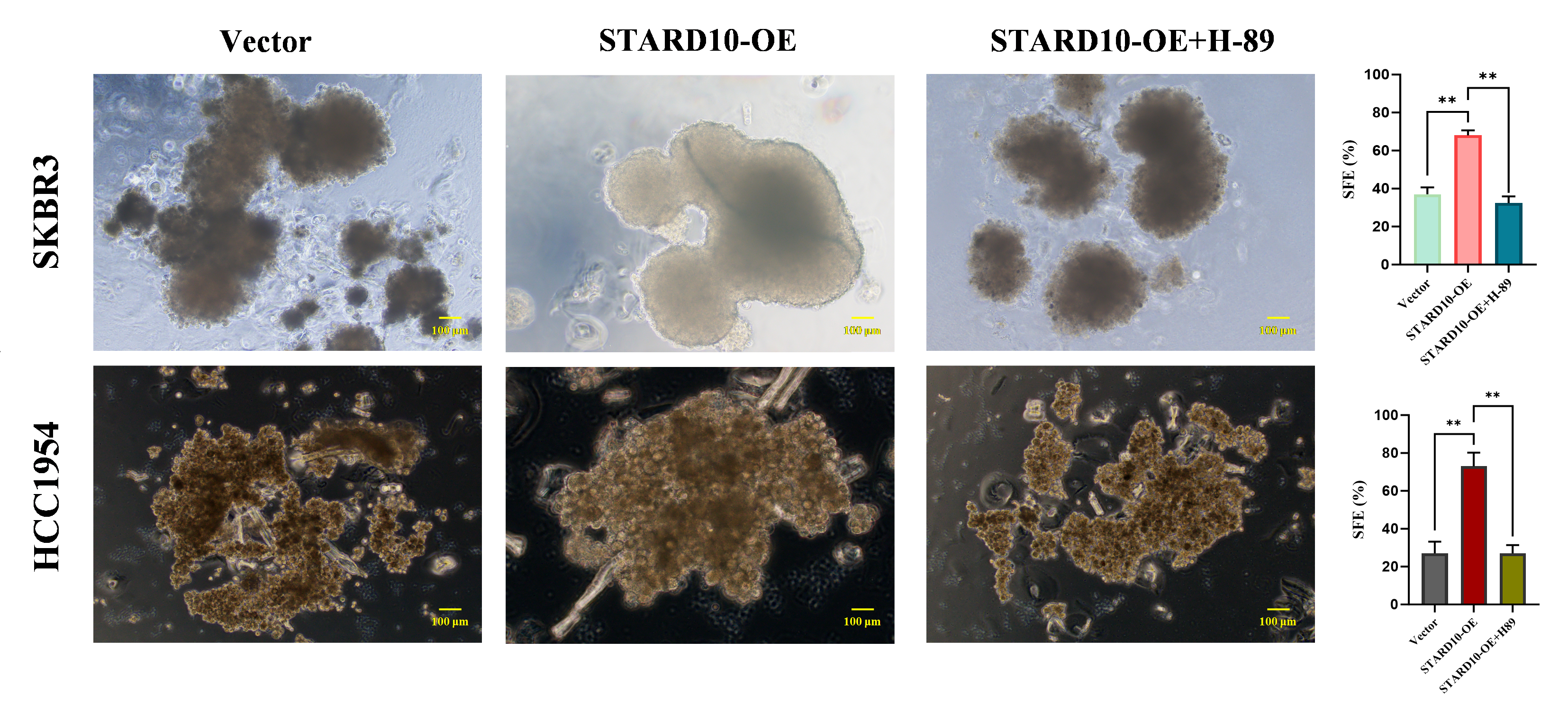


The spheroid-forming capacity of SKBR3 and HCC1954 cells stably overexpressing STARD10 was assessed using a three-dimensional spheroid formation assay following treatment with the H-89 inhibitor. Scale bar = 100 μm. n = 3. ***P* < 0.01.

***Fig.S15* Effect of H-89 inhibitor on lipid droplets formation in STARD10-overexpressing cells.**

**
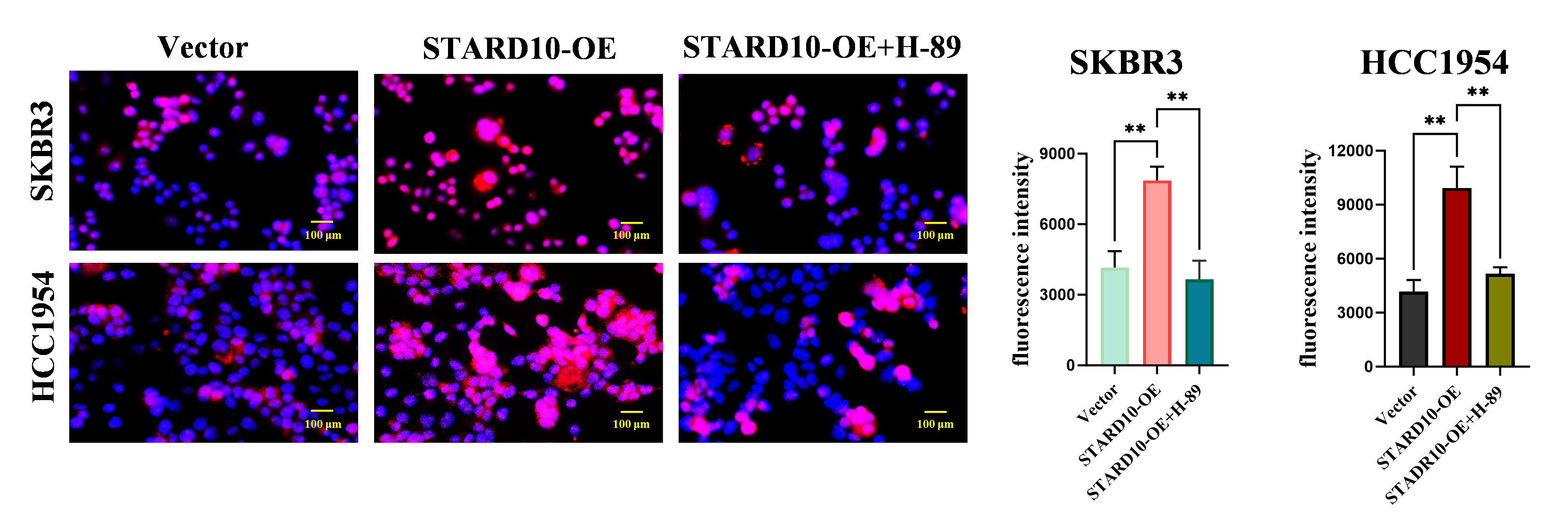
**

Representative images of intracellular lipid droplets in SKBR3 and HCC1954 cells stained with Nile Red staining following treatment with H-89. The corresponding quantification shows the fluorescence intensity of lipid droplets. Scale bar = 100 μm. n = 3. ***P* < 0.01.

***Fig.S16* Western blot analysis of FASN and DGAT1 expression after H-89 inhibitor treatment.**


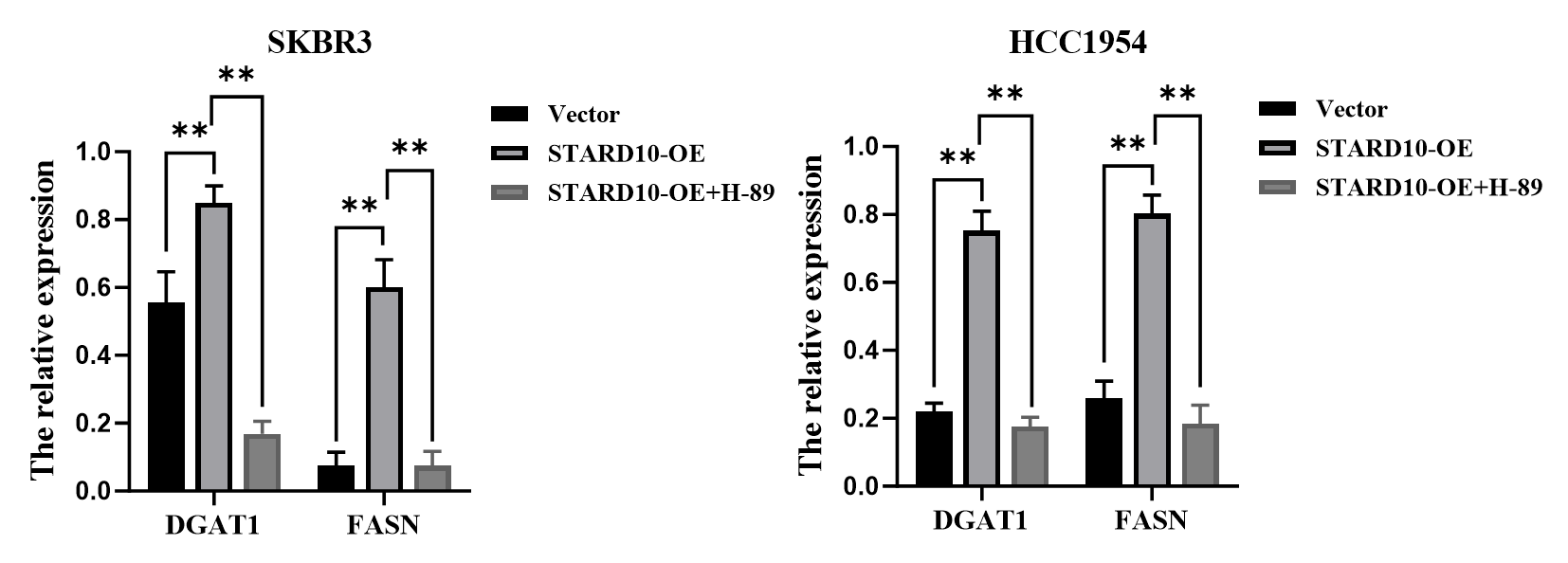


Statistical analysis of FASN and DGAT1 expression levels upon H-89 inhibitor treatment in SKBR3 and HCC1954 cells. n = 3. ***P* < 0.01.
